# Influence of body visualization in VR during the execution of motoric tasks in different age groups

**DOI:** 10.1371/journal.pone.0263112

**Published:** 2022-01-25

**Authors:** Stefan Pastel, Katharina Petri, Dan Bürger, Hendrik Marschal, Chien-Hsi Chen, Kerstin Witte

**Affiliations:** Department of Sports Engineering and Movement Science, Institute III: Sports Science, Otto-von-Guericke-University, Magdeburg, Germany; University rehabilitation institute, SLOVENIA

## Abstract

Virtual reality (VR) has become a common tool and is often considered for sport-specific purposes. Despite the increased usage, the transfer of VR-adapted skills into the real-world (RW) has not yet been sufficiently studied, and it is still unknown how much of the own body must be visible to complete motoric tasks within VR. In addition, it should be clarified whether older adults also need to perceive their body within VR scenarios to the same extent as younger people extending the usability. Therefore, younger (18–30 years old) and elderly adults (55 years and older) were tested (n = 42) performing a balance-, grasping- and throwing task in VR (HMD based) accompanied with different body visualization types in VR and in the RW having the regular visual input of body’s components. Comparing the performances between the age groups, the time for completion, the number of steps (balance task), the subjective estimation of difficulty, the number of errors, and a rating system revealing movements’ quality were considered as examined parameters. A one-way ANOVA/Friedmann with repeated measurements with factor [body visualization] was conducted to test the influence of varying body visualizations during task completion. Comparisons between the conditions [RW, VR] were performed using the t-Tests/Wilcoxon tests, and to compare both age groups [young, old], t-Tests for independent samples/Mann-Whitney-U-Test were used. The analyses of the effect of body visualization on performances showed a significant loss in movement’s quality when no body part was visualized (p < .05). This did not occur for the elderly adults, for which no influence of the body visualization on their performance could be proven. Comparing both age groups, the elderly adults performed significantly worse than the young age group in both conditions (p < .05). In VR, both groups showed longer times for completion, a higher rating of tasks’ difficulty in the balance and throwing task, and less performance quality in the grasping task. Overall, the results suggest using VR for the elderly with caution to the task demands, and the visualization of the body seemed less crucial for generating task completion. In summary, the actual task demands in VR could be successfully performed by elderly adults, even once one has to reckon with losses within movement’s quality. Although more different movements should be tested, basic elements are also realizable for elderly adults expanding possible areas of VR applications.

## 1 Introduction

Virtual reality (VR) has become a common tool to assess and treat health problems [1–3] due to the advantage of ecological validity, good experimental control, and many possibilities to track and analyze users’ behavior [4, 5]. According to [7], VR is defined as 3D environment providing stereoscopic vision and adaptive viewpoint. It is also described as a fully computer-generated environment surrounding the user [6]. Although VR is an often-used tool for sports training [7, 8] or rehearsal [5], further studies are needed to explore large scopes of tasks, sports and populations, and to better establish transfer from VR into real-world (RW) [7]. Unfortunately, the controversial results of previously conducted studies hamper the VR-application, and especially the transfer of VR-adapted skills into RW was not part of their examinations.

Not confirming transfer effects could be caused by differences in visual perception of virtual environments like underestimating distances from egocentric perspectives [9], or incongruences between sensory and motor input occurring in VR might affect the performances [4, 10]. That could affect action characteristics in VR compared to RW and impair transfer in the real world. Otherwise, previously conducted studies showed minor differences between the visual perception in RW and VR, e.g., in eye-tracking [11], spatial orientation [12–14], as well as analyses of balance, grasping, and throwing performance accompanied with different body visualization types [15]. Those rare differences conclude that VR is suitable for training applications and sports science research. Thus, it is assumable that realistic conditions are provided in VR since the user can naturally walk within the VR scene (not restricted through a seated position and hand-held controller), resulting in movement executions that are close to those in RW. However, in all those studies, only young and healthy adults were analyzed.

Considering the usage of VR for elderly adults, previous studies showed that they also can (and should) use VR technology for training and clinical applications (for state-of-the-art VR-systems for seniors, referred to [16]. [1] found that seniors, although less technology experienced, have a neutral or even positive attitude towards new technologies and only feel few problems of cybersickness (physical discomfort), which is in line with a previous study of analyzing effects of cybersickness in different age groups [17]. [18] drew the same conclusion when analyzing young and elderly adults in HMD-based VR and desktop VR. However, fatigue and perceptual overload can occur in all age groups [18]. Therefore, familiarization phases and possibilities of breaks should be implemented, and low duration of VR sessions recommended. Further investigations proved that also motoric tasks could be completed by seniors, such as balance training in VR, which might be suitable training to prevent risks of falls [19]. However, in many cases, such VR intervention was performed with semi-immersive Xbox and Wii systems [20] instead of using high immersive head.-mounted display virtual reality.

During movement execution, athletes are accustomed perceiving the whole body visually. In the context of how much of the body must be visual perceivable during task completion in VR has rarely been made. To generate the whole-body visualization within virtual environments, higher technical components are necessary, but unavailable for the private user yet. Nevertheless, previous findings already show benefits in visualizing at least parts of the virtual body. [21] pointed out that with a virtual body, perceptual illusions of body ownership can be generated in immersive VR in case of first-person-perspective (1PP). The stronger the illusion of body ownership, the greater are the behavioral change and performance in VR [21–23]. Users adapt to the virtual body even if the body is not entirely like the real body [21]. Further studies showed advantages using the 1PP and the illusory sense of body ownership and agency promotes the real bodies’ physiological reactions for the elderly [24] and could also improve gait and balance in stroke patients [25]. Concerning the importance of visual feedback of the own body during motor task completion, throwing in VR was analyzed with several body visualizations and better performance with full-user-body-visualization or visualization of the arms instead of no visualization was found [26]. That result is in line with the study of balancing [27], grasping and throwing performance [15]. Previous studies proved the feasibility of the tasks within VR environments, although [28] revealed the need of haptic feedback generating problem-free executing of grasping tasks in VR. Even more complex movements, such as dual motor tasks of catching and throwing are realizable within VR sessions [29].

Although several studies are using VR in sports science and practical training, only a few are conducted to act naturally and in sports specifically. Furthermore, only a small numbers used an HMD for VR-visualization, in which the whole body is visualized due to technological problems (for review, see [8]). In [15], the movement executions in VR of young and healthy adults within balancing, grasping, and throwing were quite like RW. To expand the target groups for possible older-aged VR users, it should be investigated whether there occur differences between the performances of younger and elderly adults within virtual environments to optimize VR applications. Furthermore, the role of the own body visualization concerning the execution of sport-motoric tasks should be specified to enable feasible VR scenarios with fewer technical components. Acting and perceiving within virtual environments by older-aged samples have been rarely made in the VR-research fields, which makes it hard to recommend this tool to its full extent. Besides, it should be clarified whether older-aged users also do not require the whole body visualization for task completion as previously determined [15].

Therefore, the previously conducted study [15] in which different motoric tasks were performed by a young age group is extended by including healthy seniors to analyze age effects during the performance of a balance task, a grasping task, and a throwing task. These tasks were chosen since balancing is a complex skill that controls weight distributions elicited through challenging daily life conditions, especially during sports. The grasping task is selected to examine whether an object’s interaction can be equal within VR. There is also the interest in whether targeted actions are realizable by letting the participants throw a virtualized ball into a virtualized goal. During the task completion, several body visualizations were provided to examine the importance of visual feedback of the own body. In comparing young and old aged groups, new participants were collected for the young age group (further described as young1) in the current study. To corroborate the results of the previous conducted study [15] and the current one, an additional comparison between the young age groups was made.

The current study has two primary aims: First, the impact of varying visualization types of the own virtual body on the performances between both age groups. It is also examined whether elderly adults need to perceive visually their own body in VR to the same extent as younger ones did and if the performance decreased when no virtual body was visualized as previously proven [15]. Second, it is necessary to check whether the motoric tasks can be performed in the same way in VR as RW (only considering the whole-body visualization) and whether seniors were affected in the same way as juniors.

The current study is classical structured, in which information of the participants and the procedure, including a description of each task, are presented. Since technical components play a crucial role within VR research, details including soft- and hardware are listed. In data analysis, the parameters for each task were explained being able to compare participants’ performances between both age groups. The statistical methods are also described step by step to ensure tracking of each goal. The results section starts with a short introduction of which graphs are assigned to the specific research goal.

## 2 Methods

The study was designed and conducted in accordance with the declaration of Helsinki. The approval of the Ethics Committee of the Otto-von-Guericke University at the Medical Faculty Hospital Magdeburg was obtained under the number 132/16.

### 2.1 Participants and experimental setup

Forty-two healthy participants were collected for this study. Twenty-one older-aged participants (63.14 ± 6.98 years, 13 female and 8 male) formed one group and twenty-one younger-aged participants (23.1 ± 3.3 years, 12 female and 9 male, further described as young2) the other one. The mean age of the young1 tested in the first study was 21±1.6 years (13 male and 8 female). No significant differences in age between both young groups are obtained (p>.05). The prerequisites for both groups were no or at least corrected visual impairment (wearing glasses under the HMD can be realized), no neuronal disorders and no physical impairments which prevent smooth execution of the motoric tasks. The older-aged group stated not having participated in VR experiments, nor did they collect any experiences in computer gaming before. All participants were informed about the aim and procedures and gave their written consent.

### 2.2 Procedure

**Fig 1:**
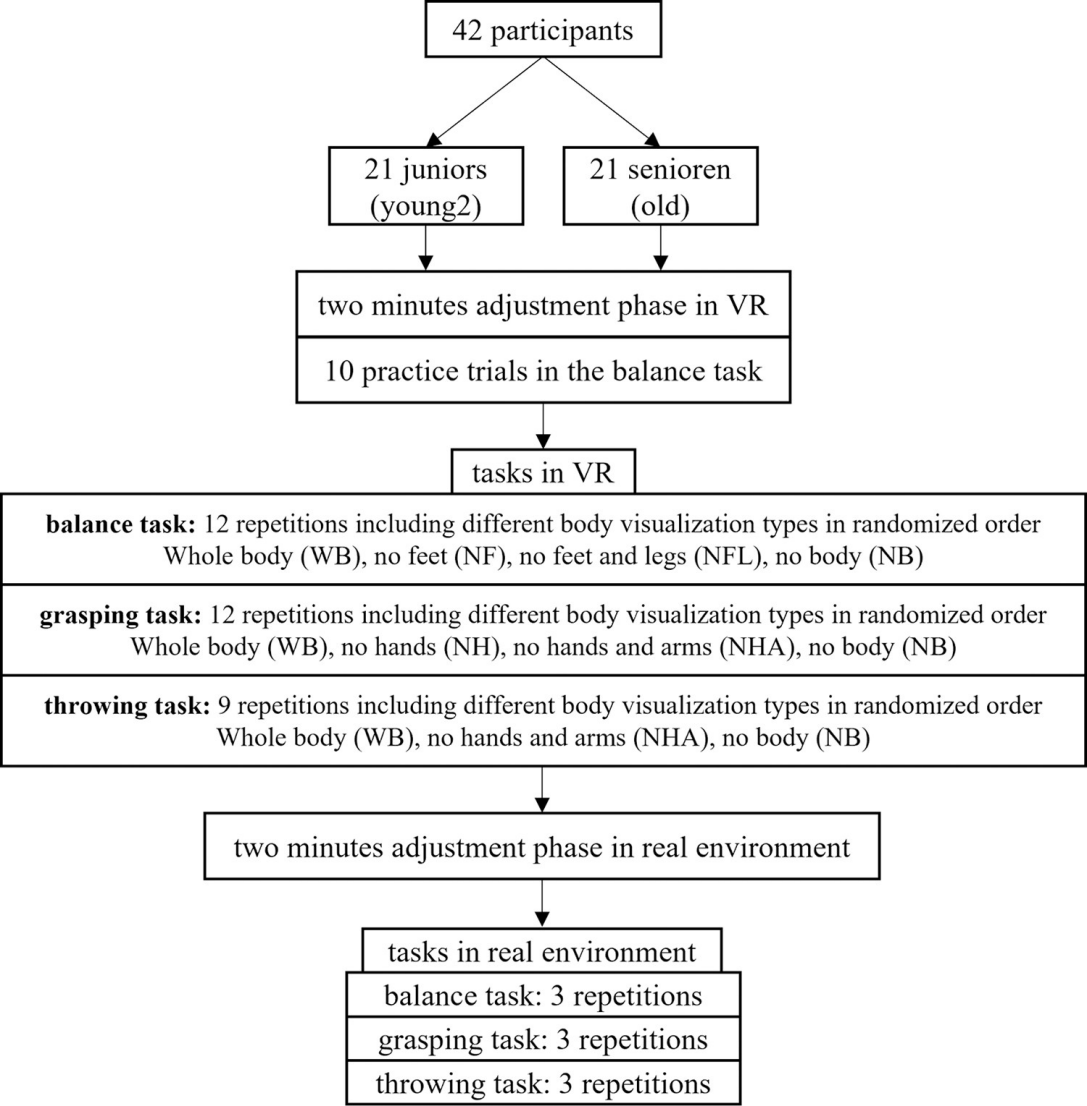
Overview of the procedure of study 2. Inspired by [33].

**Fig 2:**
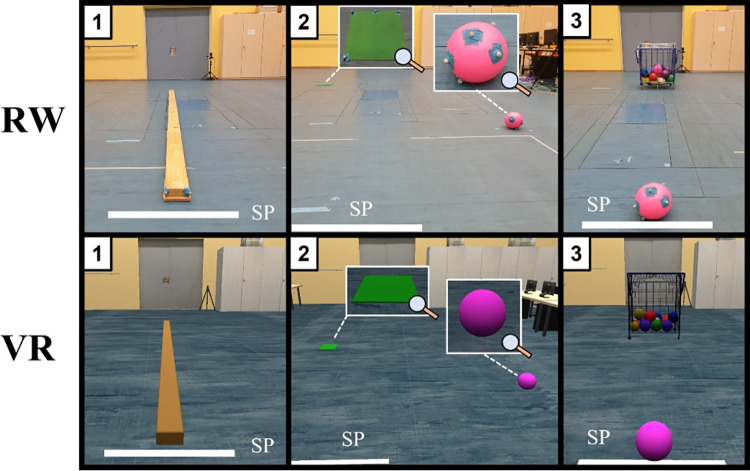
Overview of the setup of each study. The upper images series indicates the real-world setting, whereas in the bottom row the virtual room is presented. The numbers in the upper left corner represent the order of the conducted tasks in the second study (1: balance task, 2: grasping task, 3: throwing task). SP stands for starting position (also shown by the white line).

Before describing the procedure, a detailed description of each sport motoric task can be found in [15]. The participants had to conduct three motoric tasks in the VR, and afterward in real-world (RW) to ensure a within-subject comparison between the two conditions similar to [30] who started with RW first. Before performing the tasks, the participants had time first to observe the VR scene for at least three minutes. Both age groups had to start with the **balance task**, followed by the **grasping task** and the **throwing task** as the final task (see Fig 1). In the balance task, the participants were pleased to balance over the balance beam as accurately and fast as possible. The participants should pick up the ball and place it on a green pad in the grasping task. In the throwing task, the participants should throw the ball into a ball basket (see Fig 2). Each task was conducted including four different body visualization types which were the same used [15, see Fig 2]. Different visualizations were chosen whole-body (WB), without hands (NH), without the arms (NHA), without the feed (NF), without the legs (NHL), and no body part visualization. For each body visualization, three repetitions were made. After each, the participant was pleased to state the difficulty for task completion using a scale from 0 points (no difficulty) to 10 points (very difficult). All tasks were carried using a head-mounted display and a real balance beam and ball (tracked by Vicon Shogun) generalizing haptic feedback (see Fig 2). In total, the tasks’ completion in VR lasted about 15–20 minutes which is also used in other VR studies [31]. 1PP was used and a virtual body similar to RW (see [15], Fig 1) to generate a realistic perspective and increased embodiment. Afterwards, the cybersickness was also recorded using the Simulator Sickness Questionnaire (SSQ) [32].

### 2.3 Software

For modeling the virtual room, the 3D-graphic software Blender (version 2.8) was used considering the scales and the textures of the objects in the real world. For the visualization of the beam, the ball, and the goal, rigged meshes compatible with real size were used and tracked in real-time. The created virtual environments were then imported into Unity3D (version 2019.1), and the SteamVR (version 2.5.0) was used to enable users to interact in the virtual reality. Visual Studio 2017 was used for implementing the C# program for Unity to run the studies.

### 2.4 Hardware

The participants perceived the virtual environment through a head-mounted display (HTC Vive Pro, Taiwan; with a resolution of 2880 x 1600 Pixel and a field of view 110 degrees). A high-performance desktop equipped with Intel i7 CPU, 16 GB memory, 512 GB SSD, and Nvidia GTX 1080 8GB graphics card was used to ensure the presentation of VR without interruptions. A motion capture system (Vicon, Oxford, UK) including 13 cameras with a sampling rate of 200 Hz was used to visualize the own body within the VR. Two computers were connected through a network cable to track participants’ movements simultaneously to visualize those on the virtual avatar provided by Vicon Shogun. In total, 53 markers were placed on the participant’s body indicating the standard Vicon Shogun marker set. The objects which play a role within the different motoric tasks were also marked to ensure high precise position tracking in real-time and generalize haptic feedback.

### 2.5 Data analysis

First, a one-way ANOVA with repeated measurements was used to compare between the two young age groups, in which the body visualization types were treated as within subject factor and the group as between subject factor [young1, young2]. First, the performances for each task were analyzed [time for completion, number of foot strikes over the beam, number of errors, subjective estimation of difficulty] accompanied with the different body visualization types [WB, NF, NLF, NB] within each age groups [young2, old]. A detailed description of the chosen parameters is given in [15]. Hereby, a one-factor variance analysis with repeated measurement ANOVA was used with Dunn-Bonferroni-post-hoc-tests for pairwise comparisons. When no sphericity was given during the approvement of prerequisites, the Greenhouse-Geisser correction was used to interpret the results. When non-parametric data set was available, the non-parametric Friedman-test with Bonferroni-corrected post-hoc comparisons was conducted. Effect sizes were obtained using either Cohen’s formula [34] for parametric or Pearsons’ correlation coefficient (r) defined smaller as r = 0.1 small effect, r = 0.3 moderate effect, and greater than 0.5 large effect for non-parametric comparisons. Statistically significant outliers were identified by using boxplots and were then discarded. For the comparison between the conditions [RW vs. VR], a t-Test or Wilcoxon-signed rank test (for non-parametric data) was used. To determine possible significant differences between the age groups [young, old], a t-Test for independent samples or a Mann-Whitney U-test (for non-parametric data) were chosen to use. The results of the studies in the next section were processed and calculated with MATLAB R2018b. Finally, statistical analyses were performed with SPSS Version 25 (α = 0.05).

## 3 Results

The results were divided into three major parts. First, the performances of the two young age groups (young1 and young2) were compared to strengthen the expressiveness of the results from the first conducted study [15] (see Table 1). Here, no significant interactions have been found between the groups (young1 & young2) and the body visualization for all three tasks. Therefore, the body visualization had a similar impact on participants’ performances, no matter what group was tested. The resulting effects of the body visualization relate to the no body part visualization (Table 1, column titled within-subject). In addition, differences between the groups were made for each parameter and task. Except for time for completion in the balance and grasping task (obtained with small effect sizes), no significant differences between the groups were found, indicating the feasibility of the tasks.

**Table 1:** Comparisons of the performances in the motoric task between the two young age groups (young1 vs. young 2).

One-way ANOVA with repeated measurements and calculated effect sizes				
Task	Parameter	Between subject	Within-subject	Interaction effect
		Groups (young1 vs young2)	Body visualization (WB, NLF, NF, NA, NHA, NBA)	Group*Body visualization
**Balance**	time for completion	*F* (1,38) = 4.576, p = .039, effect size: 0.11, small effect	*F* (2.053, 78.024) = 20.835, p < .001, effect size: 0.38, moderate effect	*F* (2.053, 78.024) = 0.190, p = .833, no effect
	number of foot strikes	*F* (1,39) = 0.658, p = .422, no effect	*F* (2.429, 94.748) = 13.716, p < .001, effect size: 0.27, moderate effect	*F* (2.429, 94.748) = 1.533, p = .217, no effect
	number of errors	*F* (1,37) = 0.215, p = .646, no effect	*F* (2.215, 81.972) = 1.275, p = .286, no effect	*F* (2.215, 81.972) = 0.126, p = .944, no effect
	subjective estimation of difficulty	*F* (1,38) = 0.01, p > .05, no effect	*F* (2.167, 82.339) = 3.215, p = .041, effect size: 0.08, small effect	*F* (2.167, 82.339) = .001, p > .05, no effect
**Grasping**	time for completion	*F* (1,38) = 5.860, p = .02, effect size: 0.14, small effect	*F* (3, 114) = 4.359, p = .006, effect size: 0.10, small effect	*F* (3, 114) = 0.140, p = .936, no effect
	subjective estimation of difficulty	*F* (1,39) = 0.004, p = .952, no effect	*F* (1.596, 62.251) = 6.085, p = .007, effect size: 0.14, small effect	*F* (1.596, 62.251) = 0.001, p = .996, no effect
**Throwing**	quality due to score system	*F* (1,39) = 0.017, p = .898, no effect	*F* (2, 78) = 17.470, p < .001, effect size: 0.32, moderate effect	*F* (2, 78) = .008, p = .992, no effect
	subjective estimation of difficulty	*F* (1,39) = 0.149, p = .701, no effect	*F* (2, 78) = 7.475, p = .001, effect size: 0.16, small effect	*F* (2, 78) = .068, p = .934, no effect

The second goal was the investigation of the different body visualization types on both age groups (young2 vs. old, see Table 2). From the results, it emerges that the different body visualization types only harmed the young age group in their performance since no significant differences between the visualization types for the elderly occurred indicated by the ANOVA/Friedman test and following Bonferroni-corrected post-hoc comparisons. When significant differences are present, this concerns the young age group (young2), mostly when no body part was visualized.

**Table 2:** Comparisons within each age group (young2 vs. old) of the different body visualization types (WB–whole body; NF–no feet; NH–no hand; NFL–no feet and leg; NHA–no hands and arms; NB–no body).

	Parameter	Age group	WB (M±SD)	NF (M±SD)	NFL (M±SD)	NB (M±SD)	Significance within the body visualization types using Friedman tests/ANOVA	Dunn-Bonferroni-post-hoc-tests
**Balance task**	time for completion (n = 21)	young	5.03 ±1.40	4.89 ± 1.01	5.04 ± 1.21	5.87 ± 1.64	*F* (1.94, 38.87) = 10.932, p < .001, effect size: 0.74, large effect	NB–WB (p = .03)
								NB–NF (p < .01)
								NB–NFL (p < .01)
		old	6.72 ± 2.81	6.59 ±2.90	6.58 ± 2.72	6.66 ± 2.90	*F* (3, 60) = 0.237, p = .870, no effect	-
	number of foot strikes (n = 21)	young	9.76 ± 1.25	9.86 ± 1.08	9.89 ± 1.19	10.30 ± 1.24	χ2 (3) = 14.848, p = .002	NB–WB (p = .006), small effect (0.29)
								NB–NF (p = .036), small effect (0.24)
		old	10.61 ± 1.51	10.39 ± 1.62	10.61 ± 1.52	10.68 ± 1.66	*F* (3, 60) = 1.474, p = .231, no effect	-
	number of errors (n = 21)	young	0.25 ± 0.35	0.22 ± 0.30	0.18 ± 0.39	0.29 ± 0.45	χ2 (3) = 4.649, p = .199, no effect	-
		old	0.77 ± 0.56	0.48 ± 0.63	0.71 ± 0.46	0.76 ± 0.48	χ2 (3) = 10.293, p = .016*	-
	subjective estimation of difficulty (n = 20)	young	3.58 ± 1.43	3.65 ± 1.82	3.48 ± 1.80	3.98 ± 1.98	*F* (3, 57) = 0.198, p = .870, no effect	-
		old	4.10 ± 1.67	3.64 ± 1.62	3.91 ± 1.63	4.09 ± 1.47	*F* (2.22, 44.32) = 0.198, p = .225, no effect	-
	**Parameter**	**Age group**	**WB (M±SD)**	**NH (M±SD)**	**NHA (M±SD)**	**NB (M±SD)**		
**Grasping task**	time for completion (n = 21)	young	4.18 ± 0.54	4.18 ± 0.62	4.14 ± 0.54	3.98 ± 0.56	χ2 (3) = 10.837, p = .013	NB–WB (p = .014), small effect (0.26)
		old	6.25 ± 2.12	6.14 ± 2.17	6.24 ± 2.44	6.24 ± 2.51	χ2 (3) = 1.708, p = .635, no effect	-
	quality due to score system (n = 21)	young	3.44 ± 0.24	3.52 ± 0.39	3.52 ± 0.27	3.68 ± 0.32	χ2 (3) = 8.942, p = .030*	-
		old	3.67 ± 0.38	3.62 ± 0.44	3.56 ± 0.32	3.48 ± 0.39	χ2 (3) = 3.760, p = .289, no effect	-
	subjective estimation of difficulty (n = 21)	young	2.62 ± 1.33	3.00 ± 1.69	3.14 ± 1.81	3.11 ± 2.06	χ2 (3) = 6.250, p = .100, no effect	-
		old	1.93 ± 0.85	1.91 ± 0.93	1.98 ± 1.08	1.90 ± 0.97	χ2 (3) = 3.493, p = .322, no effect	-
	**Parameter**	**Age group**	**WB (M±SD)**	**NHA (M±SD)**		**NB (M±SD)**		
**Throwing task**	quality due to score system (n = 21)	young	1.70 ± 0.50	1.48 ± 0.50		1.40 ± 0.58	χ2 (3) = 6.030, p = .049*	-
		old	1.43 ± 0.56	1.41 ± 0.55		1.36 ± 0.44	χ2 (3) = 1.051, p = .591, no effect	-
	Subjective estimation of difficulty (n = 21)	young	2.90 ± 1.39	3.50 ± 1.65		3.41 ± 1.79	*F* (2, 40) = 9.482, p < .001, effect size: 0.51, large effect	NB–WB (p = .012)
								NHA–WB (p = .002)
		old	2.47 ± 1.00	2.52 ± 1.00		2.62 ± 0.96	χ2 (2) = 1.254, p = .534, no effect	-

* significant effect occurs in the Friedman-test and the none corrected p-values, but the Bonferroni-corrected post-hoc-comparisons showed no significant difference.

The third part contains the comparisons of the participants’ performances between the conditions (RW vs. VR) and the age groups (young2 vs. old) (see Table 3). Testing the differences between the conditions (dark grey column, Table 3), shows significantly worse performance in VR than in RW for both age groups with large effect sizes. Significant differences between the age groups have been found in all three tasks in RW and in VR (light grey column, Table 3). The elderly group needed more time to complete the tasks than the younger age group did. The younger age group made fewer errors in the balancing than the elderly age group in both conditions (RW and VR). The subjective estimation of task difficulty did not significantly differ between the age groups neither in RW nor in VR.

**Table 3:** Overview of the comparisons between the conditions (RW vs. VR) and between the groups (young2 vs. old).

	Parameter	Group	Condition	M + SD	Significance between the conditions (RW vs. VR) and calculated effect sizes (*r*)	Significance between the groups (young vs. old) and calculated effect sizes (*r*)	
						RW	VR
**Balance task**	time for completion (n = 21)	young	RW	3.61± 0.92	t(20) = 5.179, p<.001, large effect size (0.76)	*U* = 132.000, Z = -2.226, p=.26, moderate effect (0.34)	t(40) = -2.484, p=.010, moderate effect (0.37)
			VR	5.03 ± 1.40			
		old	RW	5.55 ± 2.62	Z(*N* = 21) = -2.763, p=.006, large effect sizes (0.63)		
			VR	6.72 ± 2.81			
	number of foot strikes (n = 21)	young	RW	8.46 ± 1.10	t(20) = 5.968, p<.001, large effect size (0.80)	t(40) = -2.834, p=.007, moderate effect (0.41)	t(40) = -1.979, p = .055, no effect
			VR	9.76 ± 1.25			
		old	RW	9.78 ± 1.82	t(20) = 4.209, p<.001, large effect size (0.69)		
			VR	10.61 ± 1.51			
	number of errors (n = 21)	young	RW	0.07 ± 0.23	Z(*N* = 21) = -2.173, p=.030, moderate effect size (0.47)	*U* = 158.000, Z = -1.975, p=.48, moderate effect (0.30)	*U* = 117.500, Z = -2.645, p=.008, moderate effect (0.41)
			VR	0.25 ± 0.35			
		old	RW	0.23 ± 0.32	Z(*N* = 21) = -3.214, p=.001, large effect size (0.70)		
			VR	0.77 ± 0.56			
	subjective estimation of difficulty (n = 21 young, n = 20 old)	young	RW	2.15 ± 1.13	Z(*N* = 20) = -3.397, p=.001, large effect size (0.76)	*U* = 190.500, Z = -0.512, p = .609, no effect	t(39) = -1.050, p = .300, no effect
			VR	3.58 ± 1.43			
		old	RW	2.17 ± 1.33	Z(*N* = 21) = -3.827, p<.001, large effect size (0.84)		
			VR	4.10 ± 1.67			
**Grasping task**	time for completion (n = 21)	young	RW	3.25 ± 0.32	Z(*N* = 21) = -4.015, p<.001, large effect size (0.88)	*U* = 31.000, Z = -4.767, p<.001, large effect (0.74)	*U* = 56.500, Z = -4.126, p<.001, large effect (0.64)
			VR	4.18 ± 0.54			
		old	RW	4.91 ± 1.55	Z(*N* = 21) = -4.015, p<.001, large effect size (0.88)		
			VR	6.25 ± 2.12			
	quality due to score system (n = 21)	young	RW	3.90 ± 0.21	Z(*N* = 21) = -4.029, p<.001, large effect size (0.88)	*U* = 208.000, Z = -0.485, p = .628, no effect	*U* = 132.500, Z = -2.254, p=.024, moderate effect (0.35)
			VR	3.44 ± 0.24			
		old	RW	3.94 ± 0.17	Z(*N* = 21) = -2.404, p=.016, large effect size (0.52)		
			VR	3.67 ± 0.38			
	subjective estimation of difficulty (n = 21)	young	RW	1.48 ± 0.68	Z(*N* = 21) = -3.672, p<.001, large effect size (0.80)	*U* = 167.000, Z = -1.543, p = .123, no effect	*U* = 146.500, Z = -1.888, p = .059, no effect
			VR	2.62 ± 1.33			
		old	RW	1.22 ± 0.41	Z(*N* = 21) = -4.029, p<.001, large effect size (0.71)		
			VR	1.93 ± 0.85			
**Throwing task**	quality due to score system (n = 21)	young	RW	1.86 ± 0.27	Z(*N* = 21) = -1.041, p = .298, no effect	*U* = 119.000, Z = -2.841, p=.004, moderate effect (0.44)	*U* = 151.500, Z = -1.827, p = .068, no effect
			VR	1.70 ± 0.50			
		old	RW	1.59 ± 0.38	Z(*N* = 21) = -0.923, p = .356, no effect		
			VR	1.43 ± 0.56			
	subjective estimation of difficulty (n = 21)	young	RW	1.98 ± 1.01	Z(*N* = 21) = -3.114, p=.002, large effect size (0.68)	*U* = 195.000, Z = -0.666, p = .505, no effect	*U* = 173.000, Z = -1.198, p = .231, no effect
			VR	2.90 ± 1.39			
		old	RW	1.63 ± 0.57	Z(*N* = 21) = -3.539, p<.001, large effect size (0.77)		
			VR	2.47 ± 1.00			

Significant differences and the condition with worse performance are marked with grey shading. M is indicating the mean and SD defined as standard deviation.

**Fig 3:**
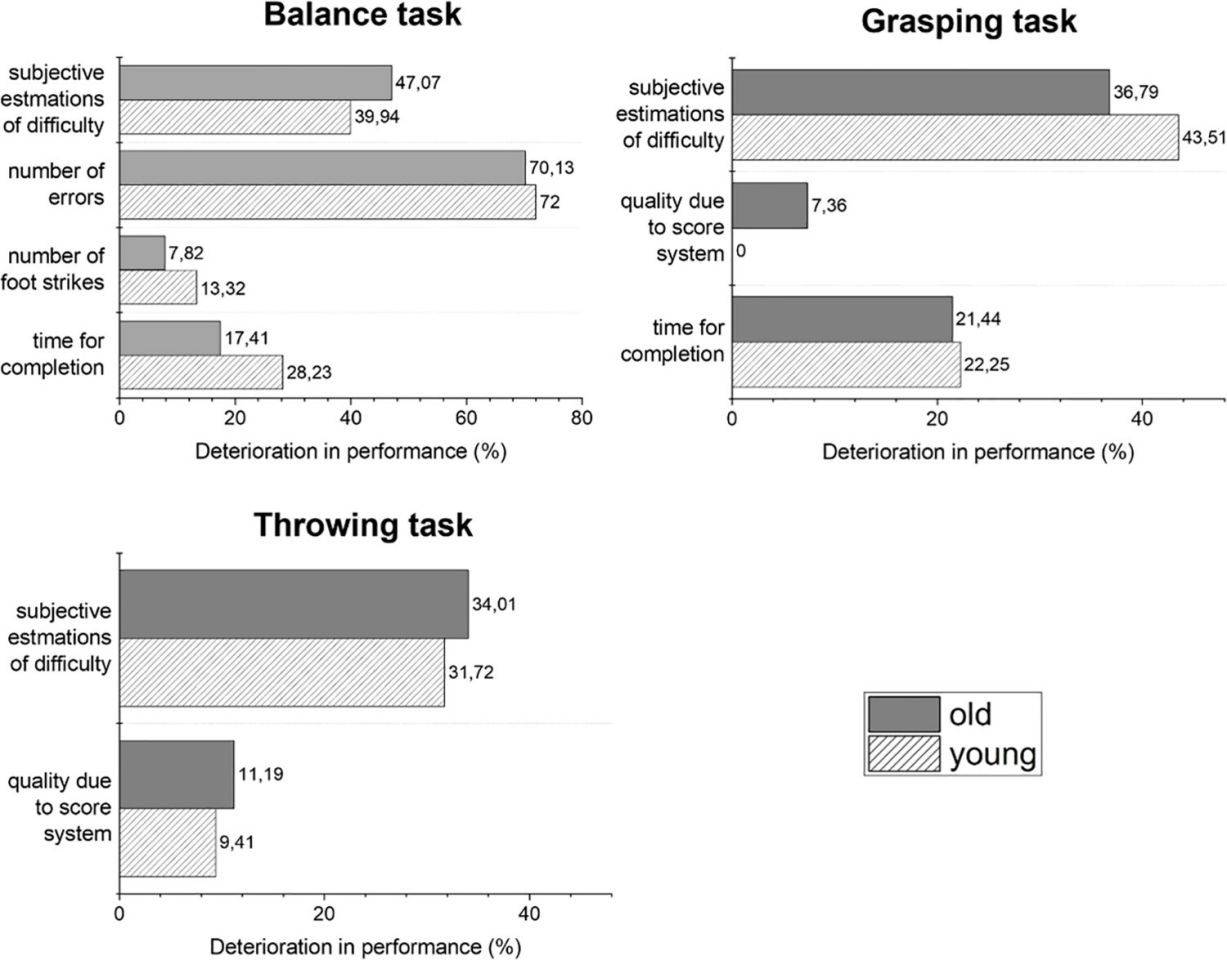
The decreased performances (in percentage) in VR (results of the whole-body visualization) for both age groups.

To show the extent of the drop of performance in VR, the percentages of RW related to the values of VR are calculated and visualized (see Fig 3). In the balance task, the drop of performance was higher for the younger adults in the number of errors, number of foot strikes, and time for completion. In the throwing task, the drop of performance was worse for the elderly aged group observable for both parameters. During the grasping task, the younger adults had no loss in the quality of throwing. Therefore, no percentage is presented. However, in percentage terms, the younger adults had a higher drop-in time for completion and subjective estimations in difficulty.

## 4 Discussion

The current article’s aim is to compare the behavior between two different age groups in the real and virtual environment. The behavior was examined by completing tasks requiring motoric skills such as balancing, grasping, and throwing. First, a comparison was made between the first [15] and the second young age group to test whether the VR performances were constantly and reliable. Afterwards, the effects of body visualization on both groups’ performances were discussed (young vs old). In the last part, the performances between RW and VR for both age groups are compared.

### 4.1 Comparison between both young age groups (young1 vs. young2)

The results indicate that both young age groups similarly performed all examined motoric tasks. Significant differences accompanied by small effect sizes occur for the time for completion in the balance and grasping task. These shifts were also observed by comparing the time duration in RW, in which the second young group also needed less time to complete the task. The quality of movement (determined by the same scoring system except for the grasping task) and the subjective estimation of difficulty do not differ between the groups, which reinforces the feasibility of the tasks.

In both groups, the body visualization’s importance turned out, since differences occurred between the performances when different types of body visualization were presented. Since no significant differences within the interactions are found [group, body visualization], it is concludable that both age young age groups needed to perceive at least one remaining limb where the body’s position could be adjusted, which is in line with previous findings [26]. This is confirmed through the worst performances for both young age groups occurred when no body part was visualized. These results suggest that participants need to have a relation to the real-body, but also shows that it is not absolutely necessary to visualize the whole-body (no significant difference between the WB, NFL, NF, NHA, NH are found). Even when no body visualization was provided, the performances are still doable with impact on movement’s quality, but still allows to complete the tasks.

### 4.2 Effects of body visualization on participants’ performances within both age groups (young2 vs. old)

The analysis of the impact of the different body visualization types on participants’ performances showed that the younger age group was most affected by the no visualization type (see Table 2). During the balance, significant differences occur between the NB condition compared to all others for the time of completion. For this task, it seemed to be important to see at least one body part, since no visualization led to a higher duration for completion (see Table 2). Similarly, more foot strikes have been taken by the participants when no body was perceivable visually, which indicates a higher degree of insecurity as already mentioned in [15]. Previous findings endorse the necessary visualization of at least one body part, since an improved step accuracy and coordination was available when full body was perceivable [35].

For the elderly aged group, there is no difference in performances affecting by the different body visualization, even if no body was visual perceivable. They were not affected by the different visualization types which are consisted of their verbal feedback, since in some cases, participants from this group stated not having realized the missing visualizations. This could be reasoned by the difference in attention on the task demands or on the limited Field of View (FoV), which is also discussed in previous research [12, 15]. In addition, in an advanced age, the visual information processing decrease [36] and they rely more on the proprioceptive input [37], which could lead to a loss in paying visual attention to the own body parts.

For both age groups, no significant differences were found in the number of errors and the subjective estimation of difficulty. Accordingly, the difficulty of each task did not increase over an increased reduction of body visualization types. The younger adults were more sensitive according to the perception of their own body, since they experienced a strange feeling when no visualization was provided. Previous studies could show that the visualization of a realistic looking avatar lead to better performances and an increased feeling of being present indicated by a more naturalistic interaction, improved spatial perception, more precise distance estimation and less cognitive load [38–40]. If the elderly adult’s attention was driven to other cues in the virtual environment, those benefits of possessing an own virtual body could not have been used.

Considering the grasping task, a significant difference occurred only within the younger aged group. The best performances mirrored by the shortest duration of grasping the ball and placing it to the target position was observed when no body was visualized. The participants verbalized confusion since the hands of the virtual body were merged sometimes with the mesh of the ball, which could have had an effect of maintaining their sense of presence [41]. This led to a feeling of grasping through the ball, which could lead to worse performances due to incongruence of the visual and haptic feedback. That was also well discussed in [41], which could show that multimodal feedback improves overall task performances. For the elderly group, the same behavior was observable compared to the balance task previously, since no significant impact of body visualization types on participants’ performances was crystallized. The quality of performances did not differ significantly in all three tasks. In addition, the subjective estimation of difficulty remained the same, which was ascertained overall tasks for each individual visualization.

The results of the throwing tasks confirmed the previously investigated findings. No quality loss within the performance of the participants was found, which is not in line with previous findings which revealed decreased accuracy in hitting a target by throwing a ball in VR [7]. The younger aged group seemed to be more sensitive facing the different virtual body parts again, since during the whole-body visualization, the task was estimated easier compared to the other visualization types. Previous findings showed that during throwing tasks, the pursuit of the own arm movement is supportive concerning the own confidence [42], however, this was not confirmed in our current findings.

Many of the presented findings coincide with previous results, which show that the visualization of the hands and feet were sufficient to generate a feeling of owning a virtual body [43]. Further studies also revealed that the whole body visualization could be a hindrance during task completion [23, 44]. During the current study, the participants received visual, auditory, and haptic feedback while conducting the tasks. The whole body was visualized and the objects that were used by the participants to complete each task were modeled and marked to track the position in real-time to ensure haptic feedback in VR. To virtualize the whole-body visualization, a whole set of hardware is required to realize research or the sport motoric tasks, which harm the transmission into practical use.

### 4.3 Differences between the condition (RW vs. VR) and the age groups (young2 vs. old)

Regarding the results presented in Table 3, significant differences between RW and VR occurred in most parameters distributed over the different tasks, which is in line with previous findings [10, 45]. Those differences can be explained through underestimations in distance perceptions or size properties [46], instability of the gait [47], awareness of objects’ position within the virtual environment [48] or reduced movement velocities [49]. Exceptions were found during the throwing task through no significant differences between the conditions within participants’ performances investigated through the scoring system. This task could be done at the same performance level in both environments; even it was rated more difficult in VR. In both other tasks, the performances were worse in VR compared to RW, the participants needed significantly longer to complete the tasks, rated the tasks more difficult and in the balance task, they also revealed a higher number of foot strikes on the beam (could be a sign for more insecurity) and more failures (stepping from the beam) were captured. This represents the performances of both age groups, which reveals the possible difficulty in performances due to the specific task demands and not caused by the different environments. Unusual results are given in the grasping task, as according to scoring system, the performances in RW are worse compared to those from VR in both age groups. One part to determine the quality in this task was to rate the end position of the placed ball, which should be in the center of the target area. Since the performances in RW were faster compared to those in VR, a higher inaccuracy of the placed ball within the target area can be explained by higher velocity hampering a precise placement of the ball.

Further comparisons between the younger and the older-aged group within both environments were made. The differences between the groups are not surprising. It is noticeable that the older- group took longer to master the balancing and the grasping task in both conditions (p < .05). In the balance task, further restrictions such as higher number of foot strikes and errors were identified in the older-aged group under real conditions. In VR, the number of foot strikes did not differ compared to the younger aged group. One main factor that could be responsible for the founded differences between the groups is the familiarity with VR systems, since the participants within the older-aged groups experienced the virtual environment for the first time. Previous studies showed the importance of adjustment phases within virtual environments which could lead to better performances [50].

The study revealed significant worse performances of the elderly compared to the younger aged group in RW as well as in VR. The tasks were obviously more difficult to complete in VR except the throwing task, although it was higher rated in the subjective difficulty in both groups. In addition, the different types of visualization of the body parts had no impact on the performances of the older-aged group. In contrast, the younger aged group seemed to be more sensitive to the visual perception of different body parts, since significant worse performances was observable when the body was not visualized at all. The advantages of the visualization of the own body on acting or performing in VR were crystallized in other examinations [15, 35, 51]. In the current study, the importance perceiving the own body is not given for older-aged people, which could rely on the reduced meaning of visual information with increasing age [36]. It can be concluded that training with seniors in VR does not absolutely require the own body visualization, which simplifies the application. To amplify this assumption, more research in this field of “how much must be visualized of the own body in VR” especially for seniors must be conducted to fill this still remaining gap. The significant differences appeared in VR to a negative extent, but in small aftermaths. It should be emphasized that it depends on the tasks and the application field, whether VR for seniors is recommendable to use it to the full extent. The additional comparison to the first young age group’s performances shows that young people pay attention to the different body visualization types, especially when no body is presented. Hereby, the participants performed worse compared to the others. Expect a small number of aspects (time for completion), no significant differences are found. Even though the differences indicate small effect sizes, which endorses the constancy of task completion within VR.

It was further analyzed whether cybersickness symptoms occurred for both age groups to examine the possible use of future VR devices for different aging groups. Interestingly, the older-aged group reached a score under the threshold (20) to a “bad simulator”, whereas the young people suffered more by getting a total score of 24.58. For both groups, the disorientation was worse (juniors 29.83, seniors 25.85), whereas the nausea was less problematic (juniors 14.08, seniors 8.86). As indicated by [52], average scores above 20 are often found in previous VR studies. The authors emphasized the correct usage of the SSQ within VR studies to test the cybersickness symptoms before and after the intervention, which was not the case in the current study. They also referred to the particular use for military aviators that do not replace the average population [52]. During the experiment, the participants were continuously asked about their state of mind or health condition, and all participants did not claim any problems no matter juniors or seniors. Generally, it is recommendable using the 1PP to ensure high performance resulting from an increased feeling of being present [53] and reduces the differences to real life, which harms the risk of cybersickness.

## 5 Limitation

To expand the usage of VR for sports-related purposes, even more, different sport-motoric tasks should be completed, and further analyzes concerning the transfer into the real-world should be done. A further limitation is that no measurements of embodiment were included since it was just focused on the impact of less visual feedback of bodies’ components. However, this could have impacted the sense of body ownership, which is already discussed in [15].

## 6 Conclusion

To extend the possible age groups which can train via VR technology in the future, it is essential to check the requirements such technology needs. Overall, the results suggest using virtual reality for older aged people with caution to the task demands. In summary, all tasks could be completed except longer execution times in balancing and grasping (which persist to a maximum delay of two seconds). The balance task was hard to take, even for the younger aged group. Without including the practice trials before starting to balance over the beam, the participants felt insecure and repeatedly stepped down. For grasping and throwing, these problems disappeared. The subjective impressions of task difficulty were rated higher in VR for all tasks in both age groups, which could be reduced by increased participation within virtual environments. According to the participants’ behavior, the motivation, and the enjoyment from participating and completing each task were enormous, and the interest in the usage in the VR system itself increased. Those differences might be minimized when more adjustment of perceiving and acting in virtual environments take place or technical improvement providing more realistic visualization were made. In addition, it is not required to visualize the whole body either for the young or for the older adults. The older age group seemed to pay less attention to this part which positively affects the possible integration of today’s VR technology.

## Supporting information

S1 Data (ZIP)
