# Supplementary material for: Influence of body visualization in VR during the execution of motoric tasks in different age groups
Source: PLoS One. 2022 Jan 25;17(1):e0263112. doi: 10.1371/journal.pone.0263112 (PMC8789136; doi:10.1371/journal.pone.0263112)

BALANCIEREN FEHLER

| **Innersubjektfaktoren** | |
| --- | --- |
| Maß: MEASURE_1 | |
| Körpervisualisierung | Abhängige Variable |
| 1 | WB_Fehler |
| 2 | NF_Fehler |
| 3 | NLF_Fehler |
| 4 | NB_Fehler |

| **Zwischensubjektfaktoren** | | | |
| --- | --- | --- | --- |
|  | | Wertelabel | N |
| Gruppe | 1 | Junioren Gruppe 1 | 18 |
|  | 2 | Junioren Gruppe 2 | 21 |

| **Deskriptive Statistiken** | | | | |
| --- | --- | --- | --- | --- |
|  | Gruppe | Mittelwert | Std.-Abweichung | N |
| WB_Fehler | Junioren Gruppe 1 | ,2407 | ,42481 | 18 |
|  | Junioren Gruppe 2 | ,2498 | ,34757 | 21 |
|  | Gesamt | ,2456 | ,37992 | 39 |
| NF_Fehler | Junioren Gruppe 1 | ,2037 | ,25918 | 18 |
|  | Junioren Gruppe 2 | ,2168 | ,30322 | 21 |
|  | Gesamt | ,2108 | ,28016 | 39 |
| NLF_Fehler | Junioren Gruppe 1 | ,0926 | ,15363 | 18 |
|  | Junioren Gruppe 2 | ,1830 | ,38688 | 21 |
|  | Gesamt | ,1413 | ,30236 | 39 |
| NB_Fehler | Junioren Gruppe 1 | ,2778 | ,47486 | 18 |
|  | Junioren Gruppe 2 | ,2916 | ,45300 | 21 |
|  | Gesamt | ,2852 | ,45709 | 39 |

| **Mauchly-Test auf Sphärizität^a^** | | | | | | | |
| --- | --- | --- | --- | --- | --- | --- | --- |
| Maß: MEASURE_1 | | | | | | | |
| Innersubjekteffekt | Mauchly-W | Approx. Chi-Quadrat | df | Sig. | Epsilon^b^ | | |
|  |  |  |  |  | Greenhouse-Geisser | Huynh-Feldt | Untergrenze |
| Körpervisualisierung | ,519 | 23,437 | 5 | ,000 | ,738 | ,809 | ,333 |
| Prüft die Nullhypothese, daß sich die Fehlerkovarianz-Matrix der orthonormalisierten transformierten abhängigen Variablen proportional zur Einheitsmatrix verhält. | | | | | | | |
| a. Design: Konstanter Term + Gruppe  Innersubjektdesign: Körpervisualisierung | | | | | | | |
| b. Kann zum Korrigieren der Freiheitsgrade für die gemittelten Signifikanztests verwendet werden. In der Tabelle mit den Tests der Effekte innerhalb der Subjekte werden korrigierte Tests angezeigt. | | | | | | | |

| **Tests der Innersubjekteffekte** | | | | | | | |
| --- | --- | --- | --- | --- | --- | --- | --- |
| Maß: MEASURE_1 | | | | | | | |
| Quelle | | Quadratsumme vom Typ III | df | Mittel der Quadrate | F | Sig. | Partielles Eta-Quadrat |
| Körpervisualisierung | Sphärizität angenommen | ,453 | 3 | ,151 | 1,275 | ,286 | ,033 |
|  | Greenhouse-Geisser | ,453 | 2,215 | ,204 | 1,275 | ,286 | ,033 |
|  | Huynh-Feldt | ,453 | 2,426 | ,187 | 1,275 | ,287 | ,033 |
|  | Untergrenze | ,453 | 1,000 | ,453 | 1,275 | ,266 | ,033 |
| Körpervisualisierung * Gruppe | Sphärizität angenommen | ,045 | 3 | ,015 | ,126 | ,944 | ,003 |
|  | Greenhouse-Geisser | ,045 | 2,215 | ,020 | ,126 | ,900 | ,003 |
|  | Huynh-Feldt | ,045 | 2,426 | ,018 | ,126 | ,915 | ,003 |
|  | Untergrenze | ,045 | 1,000 | ,045 | ,126 | ,724 | ,003 |
| Fehler(Körpervisualisierung) | Sphärizität angenommen | 13,129 | 111 | ,118 |  |  |  |
|  | Greenhouse-Geisser | 13,129 | 81,972 | ,160 |  |  |  |
|  | Huynh-Feldt | 13,129 | 89,779 | ,146 |  |  |  |
|  | Untergrenze | 13,129 | 37,000 | ,355 |  |  |  |

| **Tests der Zwischensubjekteffekte** | | | | | | |
| --- | --- | --- | --- | --- | --- | --- |
| Maß: MEASURE_1 | | | | | | |
| Transformierte Variable: Mittel | | | | | | |
| Quelle | Quadratsumme vom Typ III | df | Mittel der Quadrate | F | Sig. | Partielles Eta-Quadrat |
| Konstanter Term | 7,472 | 1 | 7,472 | 41,462 | ,000 | ,528 |
| Gruppe | ,039 | 1 | ,039 | ,215 | ,646 | ,006 |
| Fehler | 6,668 | 37 | ,180 |  |  |  |

| **Paarweise Vergleiche** | | | | | | |
| --- | --- | --- | --- | --- | --- | --- |
| Maß: MEASURE_1 | | | | | | |
| (I)Körpervisualisierung | (J)Körpervisualisierung | Mittlere Differenz (I-J) | Standard Fehler | Sig.^a^ | 95% Konfidenzintervall für die Differenz^a^ | |
|  |  |  |  |  | Untergrenze | Obergrenze |
| 1 | 2 | ,035 | ,055 | 1,000 | -,119 | ,189 |
|  | 3 | ,107 | ,078 | 1,000 | -,109 | ,324 |
|  | 4 | -,039 | ,094 | 1,000 | -,301 | ,222 |
| 2 | 1 | -,035 | ,055 | 1,000 | -,189 | ,119 |
|  | 3 | ,072 | ,058 | 1,000 | -,090 | ,235 |
|  | 4 | -,074 | ,095 | 1,000 | -,338 | ,189 |
| 3 | 1 | -,107 | ,078 | 1,000 | -,324 | ,109 |
|  | 2 | -,072 | ,058 | 1,000 | -,235 | ,090 |
|  | 4 | -,147 | ,080 | ,446 | -,370 | ,076 |
| 4 | 1 | ,039 | ,094 | 1,000 | -,222 | ,301 |
|  | 2 | ,074 | ,095 | 1,000 | -,189 | ,338 |
|  | 3 | ,147 | ,080 | ,446 | -,076 | ,370 |
| Basiert auf den geschätzten Randmitteln | | | | | | |
| a. Anpassung für Mehrfachvergleiche: Bonferroni. | | | | | | |

| **3. Gruppe * Körpervisualisierung** | | | | | |
| --- | --- | --- | --- | --- | --- |
| Maß: MEASURE_1 | | | | | |
| Gruppe | Körpervisualisierung | Mittelwert | Standard Fehler | 95%-Konfidenzintervall | |
|  |  |  |  | Untergrenze | Obergrenze |
| Junioren Gruppe 1 | 1 | ,241 | ,091 | ,057 | ,425 |
|  | 2 | ,204 | ,067 | ,068 | ,339 |
|  | 3 | ,093 | ,071 | -,052 | ,237 |
|  | 4 | ,278 | ,109 | ,057 | ,499 |
| Junioren Gruppe 2 | 1 | ,250 | ,084 | ,080 | ,420 |
|  | 2 | ,217 | ,062 | ,091 | ,342 |
|  | 3 | ,183 | ,066 | ,049 | ,317 |
|  | 4 | ,292 | ,101 | ,087 | ,496 |


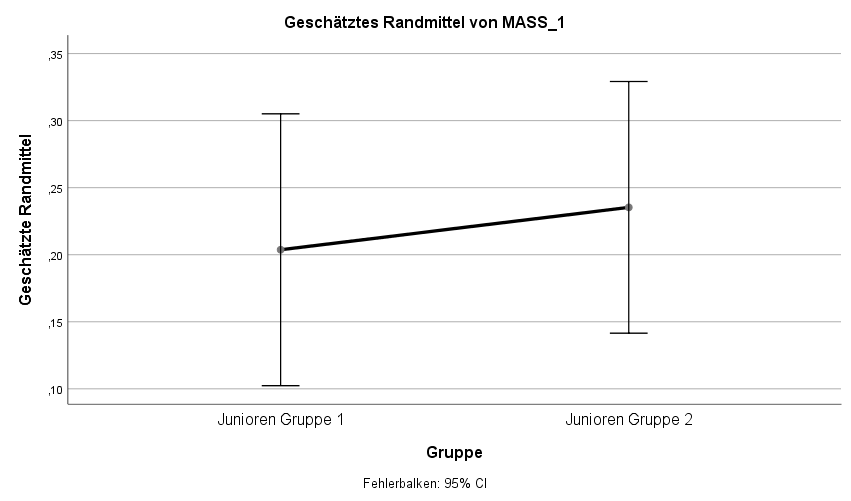

Supplement: S1 Data — (ZIP) [file pone.0263112.s001.zip › Data/Young1vsYoung2/Balancieren/BALANCIEREN FEHLER.docx]
