# Supplementary material for: Influence of body visualization in VR during the execution of motoric tasks in different age groups
Source: PLoS One. 2022 Jan 25;17(1):e0263112. doi: 10.1371/journal.pone.0263112 (PMC8789136; doi:10.1371/journal.pone.0263112)

Balancieren SE

| **Innersubjektfaktoren** | |
| --- | --- |
| Maß: MEASURE_1 | |
| Körpervisualisierung | Abhängige Variable |
| 1 | WB_SE |
| 2 | NF_SE |
| 3 | NLF_SE |
| 4 | NB_SE |

| **Zwischensubjektfaktoren** | | | |
| --- | --- | --- | --- |
|  | | Wertelabel | N |
| Gruppe | 1 | Junioren Gruppe 1 | 20 |
|  | 2 | Junioren Gruppe 2 | 20 |

| **Deskriptive Statistiken** | | | | |
| --- | --- | --- | --- | --- |
|  | Gruppe | Mittelwert | Std.-Abweichung | N |
| WB_SE | Junioren Gruppe 1 | 3,5833 | 1,43423 | 20 |
|  | Junioren Gruppe 2 | 3,5833 | 1,43423 | 20 |
|  | Gesamt | 3,5833 | 1,41572 | 40 |
| NF_SE | Junioren Gruppe 1 | 3,6500 | 1,81764 | 20 |
|  | Junioren Gruppe 2 | 3,6500 | 1,81764 | 20 |
|  | Gesamt | 3,6500 | 1,79418 | 40 |
| NLF_SE | Junioren Gruppe 1 | 3,4833 | 1,80472 | 20 |
|  | Junioren Gruppe 2 | 3,4833 | 1,80472 | 20 |
|  | Gesamt | 3,4833 | 1,78143 | 40 |
| NB_SE | Junioren Gruppe 1 | 3,9833 | 1,98378 | 20 |
|  | Junioren Gruppe 2 | 3,9833 | 1,98378 | 20 |
|  | Gesamt | 3,9833 | 1,95818 | 40 |

| **Mauchly-Test auf Sphärizität^a^** | | | | | | | |
| --- | --- | --- | --- | --- | --- | --- | --- |
| Maß: MEASURE_1 | | | | | | | |
| Innersubjekteffekt | Mauchly-W | Approx. Chi-Quadrat | df | Sig. | Epsilon^b^ | | |
|  |  |  |  |  | Greenhouse-Geisser | Huynh-Feldt | Untergrenze |
| Körpervisualisierung | ,548 | 22,071 | 5 | ,001 | ,722 | ,788 | ,333 |
| Prüft die Nullhypothese, daß sich die Fehlerkovarianz-Matrix der orthonormalisierten transformierten abhängigen Variablen proportional zur Einheitsmatrix verhält. | | | | | | | |
| a. Design: Konstanter Term + Gruppe  Innersubjektdesign: Körpervisualisierung | | | | | | | |
| b. Kann zum Korrigieren der Freiheitsgrade für die gemittelten Signifikanztests verwendet werden. In der Tabelle mit den Tests der Effekte innerhalb der Subjekte werden korrigierte Tests angezeigt. | | | | | | | |

| **Tests der Innersubjekteffekte** | | | | | | | |
| --- | --- | --- | --- | --- | --- | --- | --- |
| Maß: MEASURE_1 | | | | | | | |
| Quelle | | Quadratsumme vom Typ III | df | Mittel der Quadrate | F | Sig. | Partielles Eta-Quadrat |
| Körpervisualisierung | Sphärizität angenommen | 5,633 | 3 | 1,878 | 3,215 | ,026 | ,078 |
|  | Greenhouse-Geisser | 5,633 | 2,167 | 2,600 | 3,215 | ,041 | ,078 |
|  | Huynh-Feldt | 5,633 | 2,363 | 2,384 | 3,215 | ,037 | ,078 |
|  | Untergrenze | 5,633 | 1,000 | 5,633 | 3,215 | ,081 | ,078 |
| Körpervisualisierung * Gruppe | Sphärizität angenommen | ,000 | 3 | ,000 | ,000 | 1,000 | ,000 |
|  | Greenhouse-Geisser | ,000 | 2,167 | ,000 | ,000 | 1,000 | ,000 |
|  | Huynh-Feldt | ,000 | 2,363 | ,000 | ,000 | 1,000 | ,000 |
|  | Untergrenze | ,000 | 1,000 | ,000 | ,000 | 1,000 | ,000 |
| Fehler(Körpervisualisierung) | Sphärizität angenommen | 66,589 | 114 | ,584 |  |  |  |
|  | Greenhouse-Geisser | 66,589 | 82,339 | ,809 |  |  |  |
|  | Huynh-Feldt | 66,589 | 89,793 | ,742 |  |  |  |
|  | Untergrenze | 66,589 | 38,000 | 1,752 |  |  |  |

| **Tests der Zwischensubjekteffekte** | | | | | | |
| --- | --- | --- | --- | --- | --- | --- |
| Maß: MEASURE_1 | | | | | | |
| Transformierte Variable: Mittel | | | | | | |
| Quelle | Quadratsumme vom Typ III | df | Mittel der Quadrate | F | Sig. | Partielles Eta-Quadrat |
| Konstanter Term | 2160,900 | 1 | 2160,900 | 200,067 | ,000 | ,840 |
| Gruppe | ,000 | 1 | ,000 | ,000 | 1,000 | ,000 |
| Fehler | 410,433 | 38 | 10,801 |  |  |  |

| **Paarweise Vergleiche** | | | | | | |
| --- | --- | --- | --- | --- | --- | --- |
| Maß: MEASURE_1 | | | | | | |
| (I)Körpervisualisierung | (J)Körpervisualisierung | Mittlere Differenz (I-J) | Standard Fehler | Sig.^b^ | 95% Konfidenzintervall für die Differenz^b^ | |
|  |  |  |  |  | Untergrenze | Obergrenze |
| 1 | 2 | -,067 | ,176 | 1,000 | -,556 | ,422 |
|  | 3 | ,100 | ,209 | 1,000 | -,483 | ,683 |
|  | 4 | -,400 | ,214 | ,416 | -,996 | ,196 |
| 2 | 1 | ,067 | ,176 | 1,000 | -,422 | ,556 |
|  | 3 | ,167 | ,118 | ,993 | -,161 | ,495 |
|  | 4 | -,333 | ,152 | ,207 | -,756 | ,090 |
| 3 | 1 | -,100 | ,209 | 1,000 | -,683 | ,483 |
|  | 2 | -,167 | ,118 | ,993 | -,495 | ,161 |
|  | 4 | -,500^*^ | ,133 | ,003 | -,870 | -,130 |
| 4 | 1 | ,400 | ,214 | ,416 | -,196 | ,996 |
|  | 2 | ,333 | ,152 | ,207 | -,090 | ,756 |
|  | 3 | ,500^*^ | ,133 | ,003 | ,130 | ,870 |
| Basiert auf den geschätzten Randmitteln | | | | | | |
| *. Die mittlere Differenz ist auf dem ,05-Niveau signifikant. | | | | | | |
| b. Anpassung für Mehrfachvergleiche: Bonferroni. | | | | | | |

| **3. Gruppe * Körpervisualisierung** | | | | | |
| --- | --- | --- | --- | --- | --- |
| Maß: MEASURE_1 | | | | | |
| Gruppe | Körpervisualisierung | Mittelwert | Standard Fehler | 95%-Konfidenzintervall | |
|  |  |  |  | Untergrenze | Obergrenze |
| Junioren Gruppe 1 | 1 | 3,583 | ,321 | 2,934 | 4,233 |
|  | 2 | 3,650 | ,406 | 2,827 | 4,473 |
|  | 3 | 3,483 | ,404 | 2,666 | 4,300 |
|  | 4 | 3,983 | ,444 | 3,085 | 4,881 |
| Junioren Gruppe 2 | 1 | 3,583 | ,321 | 2,934 | 4,233 |
|  | 2 | 3,650 | ,406 | 2,827 | 4,473 |
|  | 3 | 3,483 | ,404 | 2,666 | 4,300 |
|  | 4 | 3,983 | ,444 | 3,085 | 4,881 |


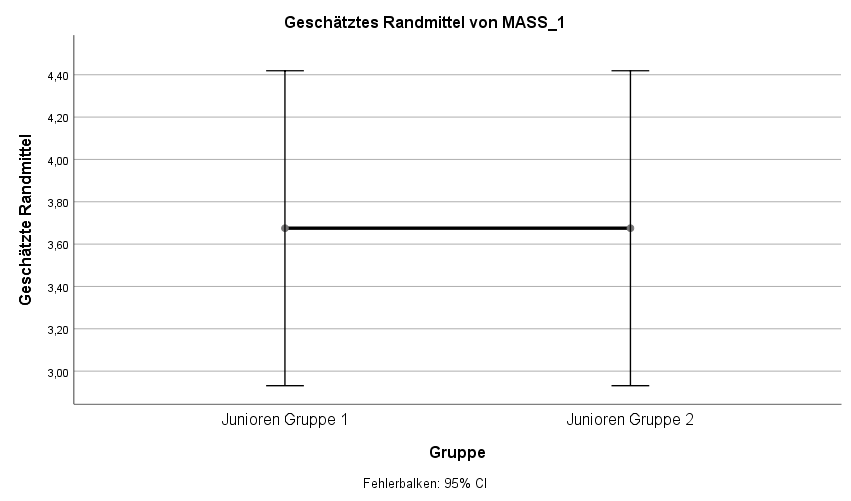

Supplement: S1 Data — (ZIP) [file pone.0263112.s001.zip › Data/Young1vsYoung2/Balancieren/Balancieren SE.docx]
