# Supplementary material for: Influence of body visualization in VR during the execution of motoric tasks in different age groups
Source: PLoS One. 2022 Jan 25;17(1):e0263112. doi: 10.1371/journal.pone.0263112 (PMC8789136; doi:10.1371/journal.pone.0263112)

BALANCIEREN ZEIT

| **Innersubjektfaktoren** | |
| --- | --- |
| Maß: MEASURE_1 | |
| Körpervisualisierung | Abhängige Variable |
| 1 | WB_Zeit |
| 2 | NF_Zeit |
| 3 | NLF_Zeit |
| 4 | NB_Zeit |

| **Zwischensubjektfaktoren** | | | |
| --- | --- | --- | --- |
|  | | Wertelabel | N |
| Gruppe | 1 | Junioren Gruppe 1 | 19 |
|  | 2 | Junioren Gruppe 2 | 21 |

| **Deskriptive Statistiken** | | | | |
| --- | --- | --- | --- | --- |
|  | Gruppe | Mittelwert | Std.-Abweichung | N |
| WB_Zeit | Junioren Gruppe 1 | 5,9282 | 1,56627 | 19 |
|  | Junioren Gruppe 2 | 5,0254 | 1,39617 | 21 |
|  | Gesamt | 5,4543 | 1,52983 | 40 |
| NF_Zeit | Junioren Gruppe 1 | 5,7958 | 1,05156 | 19 |
|  | Junioren Gruppe 2 | 4,8874 | 1,00617 | 21 |
|  | Gesamt | 5,3189 | 1,11382 | 40 |
| NLF_Zeit | Junioren Gruppe 1 | 5,7856 | 1,40798 | 19 |
|  | Junioren Gruppe 2 | 5,0368 | 1,20539 | 21 |
|  | Gesamt | 5,3925 | 1,34294 | 40 |
| NB_Zeit | Junioren Gruppe 1 | 6,8240 | 1,86484 | 19 |
|  | Junioren Gruppe 2 | 5,8679 | 1,64150 | 21 |
|  | Gesamt | 6,3221 | 1,79462 | 40 |

| **Mauchly-Test auf Sphärizität^a^** | | | | | | | |
| --- | --- | --- | --- | --- | --- | --- | --- |
| Maß: MEASURE_1 | | | | | | | |
| Innersubjekteffekt | Mauchly-W | Approx. Chi-Quadrat | df | Sig. | Epsilon^b^ | | |
|  |  |  |  |  | Greenhouse-Geisser | Huynh-Feldt | Untergrenze |
| Körpervisualisierung | ,342 | 39,364 | 5 | ,000 | ,684 | ,743 | ,333 |
| Prüft die Nullhypothese, daß sich die Fehlerkovarianz-Matrix der orthonormalisierten transformierten abhängigen Variablen proportional zur Einheitsmatrix verhält. | | | | | | | |
| a. Design: Konstanter Term + Gruppe  Innersubjektdesign: Körpervisualisierung | | | | | | | |
| b. Kann zum Korrigieren der Freiheitsgrade für die gemittelten Signifikanztests verwendet werden. In der Tabelle mit den Tests der Effekte innerhalb der Subjekte werden korrigierte Tests angezeigt. | | | | | | | |

| **Tests der Innersubjekteffekte** | | | | | | | |
| --- | --- | --- | --- | --- | --- | --- | --- |
| Maß: MEASURE_1 | | | | | | | |
| Quelle | | Quadratsumme vom Typ III | df | Mittel der Quadrate | F | Sig. | Partielles Eta-Quadrat |
| Körpervisualisierung | Sphärizität angenommen | 26,588 | 3 | 8,863 | 20,835 | ,000 | ,354 |
|  | Greenhouse-Geisser | 26,588 | 2,053 | 12,949 | 20,835 | ,000 | ,354 |
|  | Huynh-Feldt | 26,588 | 2,229 | 11,928 | 20,835 | ,000 | ,354 |
|  | Untergrenze | 26,588 | 1,000 | 26,588 | 20,835 | ,000 | ,354 |
| Körpervisualisierung * Gruppe | Sphärizität angenommen | ,243 | 3 | ,081 | ,190 | ,903 | ,005 |
|  | Greenhouse-Geisser | ,243 | 2,053 | ,118 | ,190 | ,833 | ,005 |
|  | Huynh-Feldt | ,243 | 2,229 | ,109 | ,190 | ,849 | ,005 |
|  | Untergrenze | ,243 | 1,000 | ,243 | ,190 | ,665 | ,005 |
| Fehler(Körpervisualisierung) | Sphärizität angenommen | 48,494 | 114 | ,425 |  |  |  |
|  | Greenhouse-Geisser | 48,494 | 78,024 | ,622 |  |  |  |
|  | Huynh-Feldt | 48,494 | 84,707 | ,572 |  |  |  |
|  | Untergrenze | 48,494 | 38,000 | 1,276 |  |  |  |

| **Tests der Zwischensubjekteffekte** | | | | | | |
| --- | --- | --- | --- | --- | --- | --- |
| Maß: MEASURE_1 | | | | | | |
| Transformierte Variable: Mittel | | | | | | |
| Quelle | Quadratsumme vom Typ III | df | Mittel der Quadrate | F | Sig. | Partielles Eta-Quadrat |
| Konstanter Term | 5083,841 | 1 | 5083,841 | 754,543 | ,000 | ,952 |
| Gruppe | 30,831 | 1 | 30,831 | 4,576 | ,039 | ,107 |
| Fehler | 256,030 | 38 | 6,738 |  |  |  |

| **Paarweise Vergleiche** | | | | | | |
| --- | --- | --- | --- | --- | --- | --- |
| Maß: MEASURE_1 | | | | | | |
| (I)Körpervisualisierung | (J)Körpervisualisierung | Mittlere Differenz (I-J) | Standard Fehler | Sig.^b^ | 95% Konfidenzintervall für die Differenz^b^ | |
|  |  |  |  |  | Untergrenze | Obergrenze |
| 1 | 2 | ,135 | ,132 | 1,000 | -,233 | ,504 |
|  | 3 | ,066 | ,151 | 1,000 | -,356 | ,487 |
|  | 4 | -,869^*^ | ,196 | ,000 | -1,414 | -,324 |
| 2 | 1 | -,135 | ,132 | 1,000 | -,504 | ,233 |
|  | 3 | -,070 | ,073 | 1,000 | -,272 | ,132 |
|  | 4 | -1,004^*^ | ,160 | ,000 | -1,449 | -,560 |
| 3 | 1 | -,066 | ,151 | 1,000 | -,487 | ,356 |
|  | 2 | ,070 | ,073 | 1,000 | -,132 | ,272 |
|  | 4 | -,935^*^ | ,136 | ,000 | -1,313 | -,557 |
| 4 | 1 | ,869^*^ | ,196 | ,000 | ,324 | 1,414 |
|  | 2 | 1,004^*^ | ,160 | ,000 | ,560 | 1,449 |
|  | 3 | ,935^*^ | ,136 | ,000 | ,557 | 1,313 |
| Basiert auf den geschätzten Randmitteln | | | | | | |
| *. Die mittlere Differenz ist auf dem ,05-Niveau signifikant. | | | | | | |
| b. Anpassung für Mehrfachvergleiche: Bonferroni. | | | | | | |

| **3. Gruppe * Körpervisualisierung** | | | | | |
| --- | --- | --- | --- | --- | --- |
| Maß: MEASURE_1 | | | | | |
| Gruppe | Körpervisualisierung | Mittelwert | Standard Fehler | 95%-Konfidenzintervall | |
|  |  |  |  | Untergrenze | Obergrenze |
| Junioren Gruppe 1 | 1 | 5,928 | ,339 | 5,241 | 6,615 |
|  | 2 | 5,796 | ,236 | 5,318 | 6,273 |
|  | 3 | 5,786 | ,299 | 5,179 | 6,392 |
|  | 4 | 6,824 | ,402 | 6,011 | 7,637 |
| Junioren Gruppe 2 | 1 | 5,025 | ,323 | 4,372 | 5,679 |
|  | 2 | 4,887 | ,224 | 4,433 | 5,341 |
|  | 3 | 5,037 | ,285 | 4,460 | 5,613 |
|  | 4 | 5,868 | ,382 | 5,094 | 6,641 |


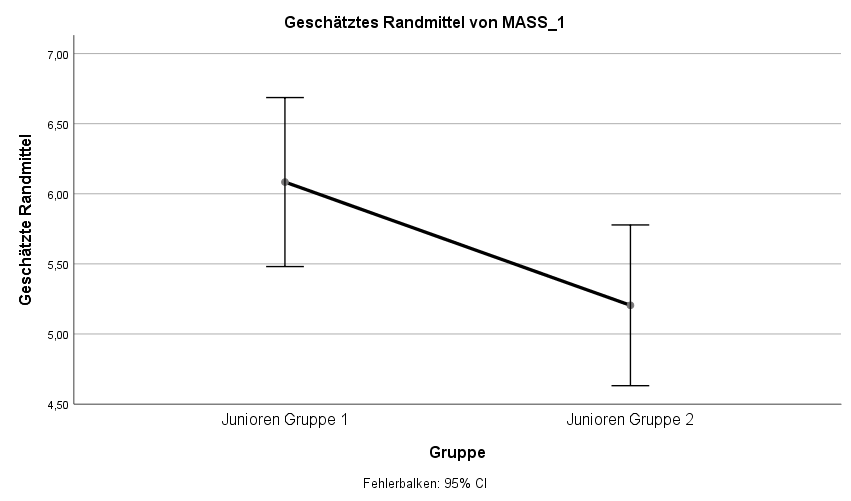

Supplement: S1 Data — (ZIP) [file pone.0263112.s001.zip › Data/Young1vsYoung2/Balancieren/BALANCIEREN ZEIT.docx]
