# Supplementary material for: Influence of body visualization in VR during the execution of motoric tasks in different age groups
Source: PLoS One. 2022 Jan 25;17(1):e0263112. doi: 10.1371/journal.pone.0263112 (PMC8789136; doi:10.1371/journal.pone.0263112)

SCHRITTE BALANCIEREN

| **Innersubjektfaktoren** | |
| --- | --- |
| Maß: MEASURE_1 | |
| Körpervisualisierung | Abhängige Variable |
| 1 | WB_Schritte |
| 2 | NF_Schritte |
| 3 | NLF_Schritte |
| 4 | NB_Schritte |

| **Zwischensubjektfaktoren** | | | |
| --- | --- | --- | --- |
|  | | Wertelabel | N |
| Gruppe | 1 | Junioren Gruppe 1 | 20 |
|  | 2 | Junioren Gruppe 2 | 21 |

| **Deskriptive Statistiken** | | | | |
| --- | --- | --- | --- | --- |
|  | Gruppe | Mittelwert | Std.-Abweichung | N |
| WB_Schritte | Junioren Gruppe 1 | 10,0333 | 1,37181 | 20 |
|  | Junioren Gruppe 2 | 9,7621 | 1,25229 | 21 |
|  | Gesamt | 9,8944 | 1,30263 | 41 |
| NF_Schritte | Junioren Gruppe 1 | 10,0333 | 1,33289 | 20 |
|  | Junioren Gruppe 2 | 9,8573 | 1,07789 | 21 |
|  | Gesamt | 9,9432 | 1,19697 | 41 |
| NLF_Schritte | Junioren Gruppe 1 | 10,1000 | 1,60080 | 20 |
|  | Junioren Gruppe 2 | 9,8887 | 1,19406 | 21 |
|  | Gesamt | 9,9918 | 1,39339 | 41 |
| NB_Schritte | Junioren Gruppe 1 | 10,9833 | 2,09838 | 20 |
|  | Junioren Gruppe 2 | 10,3016 | 1,24233 | 21 |
|  | Gesamt | 10,6341 | 1,72692 | 41 |

| **Mauchly-Test auf Sphärizität^a^** | | | | | | | |
| --- | --- | --- | --- | --- | --- | --- | --- |
| Maß: MEASURE_1 | | | | | | | |
| Innersubjekteffekt | Mauchly-W | Approx. Chi-Quadrat | df | Sig. | Epsilon^b^ | | |
|  |  |  |  |  | Greenhouse-Geisser | Huynh-Feldt | Untergrenze |
| Körpervisualisierung | ,665 | 15,393 | 5 | ,009 | ,810 | ,890 | ,333 |
| Prüft die Nullhypothese, daß sich die Fehlerkovarianz-Matrix der orthonormalisierten transformierten abhängigen Variablen proportional zur Einheitsmatrix verhält. | | | | | | | |
| a. Design: Konstanter Term + Gruppe  Innersubjektdesign: Körpervisualisierung | | | | | | | |
| b. Kann zum Korrigieren der Freiheitsgrade für die gemittelten Signifikanztests verwendet werden. In der Tabelle mit den Tests der Effekte innerhalb der Subjekte werden korrigierte Tests angezeigt. | | | | | | | |

| **Tests der Innersubjekteffekte** | | | | | | | |
| --- | --- | --- | --- | --- | --- | --- | --- |
| Maß: MEASURE_1 | | | | | | | |
| Quelle | | Quadratsumme vom Typ III | df | Mittel der Quadrate | F | Sig. | Partielles Eta-Quadrat |
| Körpervisualisierung | Sphärizität angenommen | 15,107 | 3 | 5,036 | 13,716 | ,000 | ,260 |
|  | Greenhouse-Geisser | 15,107 | 2,429 | 6,218 | 13,716 | ,000 | ,260 |
|  | Huynh-Feldt | 15,107 | 2,669 | 5,660 | 13,716 | ,000 | ,260 |
|  | Untergrenze | 15,107 | 1,000 | 15,107 | 13,716 | ,001 | ,260 |
| Körpervisualisierung * Gruppe | Sphärizität angenommen | 1,689 | 3 | ,563 | 1,533 | ,210 | ,038 |
|  | Greenhouse-Geisser | 1,689 | 2,429 | ,695 | 1,533 | ,217 | ,038 |
|  | Huynh-Feldt | 1,689 | 2,669 | ,633 | 1,533 | ,214 | ,038 |
|  | Untergrenze | 1,689 | 1,000 | 1,689 | 1,533 | ,223 | ,038 |
| Fehler(Körpervisualisierung) | Sphärizität angenommen | 42,954 | 117 | ,367 |  |  |  |
|  | Greenhouse-Geisser | 42,954 | 94,748 | ,453 |  |  |  |
|  | Huynh-Feldt | 42,954 | 104,091 | ,413 |  |  |  |
|  | Untergrenze | 42,954 | 39,000 | 1,101 |  |  |  |

| **Tests der Zwischensubjekteffekte** | | | | | | |
| --- | --- | --- | --- | --- | --- | --- |
| Maß: MEASURE_1 | | | | | | |
| Transformierte Variable: Mittel | | | | | | |
| Quelle | Quadratsumme vom Typ III | df | Mittel der Quadrate | F | Sig. | Partielles Eta-Quadrat |
| Konstanter Term | 16785,838 | 1 | 16785,838 | 2398,933 | ,000 | ,984 |
| Gruppe | 4,601 | 1 | 4,601 | ,658 | ,422 | ,017 |
| Fehler | 272,891 | 39 | 6,997 |  |  |  |

| **Paarweise Vergleiche** | | | | | | |
| --- | --- | --- | --- | --- | --- | --- |
| Maß: MEASURE_1 | | | | | | |
| (I)Körpervisualisierung | (J)Körpervisualisierung | Mittlere Differenz (I-J) | Standard Fehler | Sig.^b^ | 95% Konfidenzintervall für die Differenz^b^ | |
|  |  |  |  |  | Untergrenze | Obergrenze |
| 1 | 2 | -,048 | ,117 | 1,000 | -,374 | ,279 |
|  | 3 | -,097 | ,131 | 1,000 | -,460 | ,267 |
|  | 4 | -,745^*^ | ,162 | ,000 | -1,196 | -,293 |
| 2 | 1 | ,048 | ,117 | 1,000 | -,279 | ,374 |
|  | 3 | -,049 | ,095 | 1,000 | -,312 | ,214 |
|  | 4 | -,697^*^ | ,154 | ,000 | -1,125 | -,269 |
| 3 | 1 | ,097 | ,131 | 1,000 | -,267 | ,460 |
|  | 2 | ,049 | ,095 | 1,000 | -,214 | ,312 |
|  | 4 | -,648^*^ | ,132 | ,000 | -1,016 | -,280 |
| 4 | 1 | ,745^*^ | ,162 | ,000 | ,293 | 1,196 |
|  | 2 | ,697^*^ | ,154 | ,000 | ,269 | 1,125 |
|  | 3 | ,648^*^ | ,132 | ,000 | ,280 | 1,016 |
| Basiert auf den geschätzten Randmitteln | | | | | | |
| *. Die mittlere Differenz ist auf dem ,05-Niveau signifikant. | | | | | | |
| b. Anpassung für Mehrfachvergleiche: Bonferroni. | | | | | | |

| **3. Gruppe * Körpervisualisierung** | | | | | |
| --- | --- | --- | --- | --- | --- |
| Maß: MEASURE_1 | | | | | |
| Gruppe | Körpervisualisierung | Mittelwert | Standard Fehler | 95%-Konfidenzintervall | |
|  |  |  |  | Untergrenze | Obergrenze |
| Junioren Gruppe 1 | 1 | 10,033 | ,293 | 9,440 | 10,627 |
|  | 2 | 10,033 | ,270 | 9,487 | 10,580 |
|  | 3 | 10,100 | ,315 | 9,464 | 10,736 |
|  | 4 | 10,983 | ,383 | 10,208 | 11,758 |
| Junioren Gruppe 2 | 1 | 9,762 | ,286 | 9,183 | 10,341 |
|  | 2 | 9,857 | ,264 | 9,324 | 10,391 |
|  | 3 | 9,889 | ,307 | 9,268 | 10,510 |
|  | 4 | 10,302 | ,374 | 9,545 | 11,058 |


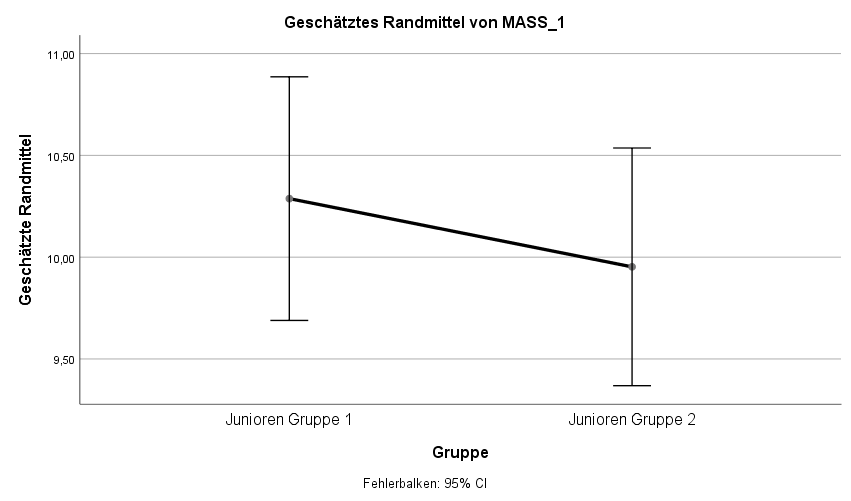

Supplement: S1 Data — (ZIP) [file pone.0263112.s001.zip › Data/Young1vsYoung2/Balancieren/SCHRITTE BALANCIEREN.docx]
