# Supplementary material for: Influence of body visualization in VR during the execution of motoric tasks in different age groups
Source: PLoS One. 2022 Jan 25;17(1):e0263112. doi: 10.1371/journal.pone.0263112 (PMC8789136; doi:10.1371/journal.pone.0263112)

GRASPING BEWERTUNG

| **Innersubjektfaktoren** | |
| --- | --- |
| Maß: MEASURE_1 | |
| Körpervisualisierung | Abhängige Variable |
| 1 | WB_SE |
| 2 | NH_SE |
| 3 | NHA_SE |
| 4 | NB_SE |

| **Zwischensubjektfaktoren** | | | |
| --- | --- | --- | --- |
|  | | Wertelabel | N |
| Gruppe | 1 | Junioren Gruppe 1 | 20 |
|  | 2 | Junioren Gruppe 2 | 21 |

| **Deskriptive Statistiken** | | | | |
| --- | --- | --- | --- | --- |
|  | Gruppe | Mittelwert | Std.-Abweichung | N |
| WB_SE | Junioren Gruppe 1 | 2,6500 | 1,35282 | 20 |
|  | Junioren Gruppe 2 | 2,6190 | 1,32617 | 21 |
|  | Gesamt | 2,6341 | 1,32247 | 41 |
| NH_SE | Junioren Gruppe 1 | 3,0333 | 1,73002 | 20 |
|  | Junioren Gruppe 2 | 2,9998 | 1,69319 | 21 |
|  | Gesamt | 3,0162 | 1,68979 | 41 |
| NHA_SE | Junioren Gruppe 1 | 3,1667 | 1,85277 | 20 |
|  | Junioren Gruppe 2 | 3,1430 | 1,80910 | 21 |
|  | Gesamt | 3,1546 | 1,80752 | 41 |
| NB_SE | Junioren Gruppe 1 | 3,1500 | 2,10395 | 20 |
|  | Junioren Gruppe 2 | 3,1110 | 2,05847 | 21 |
|  | Gesamt | 3,1300 | 2,05467 | 41 |

| **Mauchly-Test auf Sphärizität^a^** | | | | | | | |
| --- | --- | --- | --- | --- | --- | --- | --- |
| Maß: MEASURE_1 | | | | | | | |
| Innersubjekteffekt | Mauchly-W | Approx. Chi-Quadrat | df | Sig. | Epsilon^b^ | | |
|  |  |  |  |  | Greenhouse-Geisser | Huynh-Feldt | Untergrenze |
| Körpervisualisierung | ,239 | 53,915 | 5 | ,000 | ,532 | ,565 | ,333 |
| Prüft die Nullhypothese, daß sich die Fehlerkovarianz-Matrix der orthonormalisierten transformierten abhängigen Variablen proportional zur Einheitsmatrix verhält. | | | | | | | |
| a. Design: Konstanter Term + Gruppe  Innersubjektdesign: Körpervisualisierung | | | | | | | |
| b. Kann zum Korrigieren der Freiheitsgrade für die gemittelten Signifikanztests verwendet werden. In der Tabelle mit den Tests der Effekte innerhalb der Subjekte werden korrigierte Tests angezeigt. | | | | | | | |

| **Tests der Innersubjekteffekte** | | | | | | | |
| --- | --- | --- | --- | --- | --- | --- | --- |
| Maß: MEASURE_1 | | | | | | | |
| Quelle | | Quadratsumme vom Typ III | df | Mittel der Quadrate | F | Sig. | Partielles Eta-Quadrat |
| Körpervisualisierung | Sphärizität angenommen | 7,123 | 3 | 2,374 | 6,085 | ,001 | ,135 |
|  | Greenhouse-Geisser | 7,123 | 1,596 | 4,463 | 6,085 | ,007 | ,135 |
|  | Huynh-Feldt | 7,123 | 1,696 | 4,200 | 6,085 | ,006 | ,135 |
|  | Untergrenze | 7,123 | 1,000 | 7,123 | 6,085 | ,018 | ,135 |
| Körpervisualisierung * Gruppe | Sphärizität angenommen | ,001 | 3 | ,000 | ,001 | 1,000 | ,000 |
|  | Greenhouse-Geisser | ,001 | 1,596 | ,001 | ,001 | ,996 | ,000 |
|  | Huynh-Feldt | ,001 | 1,696 | ,001 | ,001 | ,997 | ,000 |
|  | Untergrenze | ,001 | 1,000 | ,001 | ,001 | ,974 | ,000 |
| Fehler(Körpervisualisierung) | Sphärizität angenommen | 45,651 | 117 | ,390 |  |  |  |
|  | Greenhouse-Geisser | 45,651 | 62,251 | ,733 |  |  |  |
|  | Huynh-Feldt | 45,651 | 66,151 | ,690 |  |  |  |
|  | Untergrenze | 45,651 | 39,000 | 1,171 |  |  |  |

| **Paarweise Vergleiche** | | | | | | |
| --- | --- | --- | --- | --- | --- | --- |
| Maß: MEASURE_1 | | | | | | |
| (I)Körpervisualisierung | (J)Körpervisualisierung | Mittlere Differenz (I-J) | Standard Fehler | Sig.^b^ | 95% Konfidenzintervall für die Differenz^b^ | |
|  |  |  |  |  | Untergrenze | Obergrenze |
| 1 | 2 | -,382 | ,142 | ,062 | -,776 | ,012 |
|  | 3 | -,520^*^ | ,159 | ,013 | -,961 | -,079 |
|  | 4 | -,496 | ,203 | ,114 | -1,059 | ,067 |
| 2 | 1 | ,382 | ,142 | ,062 | -,012 | ,776 |
|  | 3 | -,138 | ,091 | ,810 | -,390 | ,113 |
|  | 4 | -,114 | ,115 | 1,000 | -,433 | ,205 |
| 3 | 1 | ,520^*^ | ,159 | ,013 | ,079 | ,961 |
|  | 2 | ,138 | ,091 | ,810 | -,113 | ,390 |
|  | 4 | ,024 | ,081 | 1,000 | -,201 | ,250 |
| 4 | 1 | ,496 | ,203 | ,114 | -,067 | 1,059 |
|  | 2 | ,114 | ,115 | 1,000 | -,205 | ,433 |
|  | 3 | -,024 | ,081 | 1,000 | -,250 | ,201 |
| Basiert auf den geschätzten Randmitteln | | | | | | |
| *. Die mittlere Differenz ist auf dem ,05-Niveau signifikant. | | | | | | |
| b. Anpassung für Mehrfachvergleiche: Bonferroni. | | | | | | |

| **3. Gruppe * Körpervisualisierung** | | | | | |
| --- | --- | --- | --- | --- | --- |
| Maß: MEASURE_1 | | | | | |
| Gruppe | Körpervisualisierung | Mittelwert | Standard Fehler | 95%-Konfidenzintervall | |
|  |  |  |  | Untergrenze | Obergrenze |
| Junioren Gruppe 1 | 1 | 2,650 | ,299 | 2,044 | 3,256 |
|  | 2 | 3,033 | ,383 | 2,259 | 3,807 |
|  | 3 | 3,167 | ,409 | 2,339 | 3,995 |
|  | 4 | 3,150 | ,465 | 2,209 | 4,091 |
| Junioren Gruppe 2 | 1 | 2,619 | ,292 | 2,028 | 3,210 |
|  | 2 | 3,000 | ,373 | 2,245 | 3,755 |
|  | 3 | 3,143 | ,399 | 2,335 | 3,951 |
|  | 4 | 3,111 | ,454 | 2,193 | 4,029 |


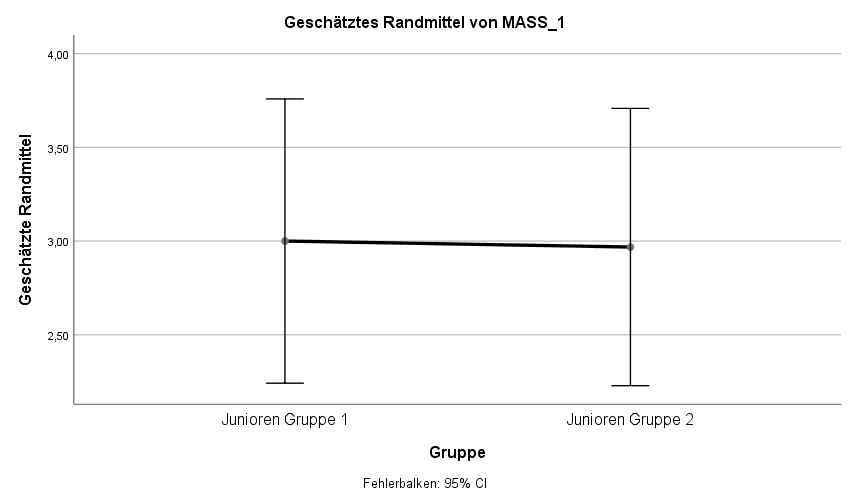

Supplement: S1 Data — (ZIP) [file pone.0263112.s001.zip › Data/Young1vsYoung2/Grasping/GRASPING BEWERTUNG.docx]
