# Supplementary material for: Influence of body visualization in VR during the execution of motoric tasks in different age groups
Source: PLoS One. 2022 Jan 25;17(1):e0263112. doi: 10.1371/journal.pone.0263112 (PMC8789136; doi:10.1371/journal.pone.0263112)

GREIFEN ZEIT

| **Innersubjektfaktoren** | |
| --- | --- |
| Maß: MEASURE_1 | |
| Körpervisualisierung | Abhängige Variable |
| 1 | WB_Zeit |
| 2 | NH_Zeit |
| 3 | NHA_Zeit |
| 4 | NB_Zeit |

| **Zwischensubjektfaktoren** | | | |
| --- | --- | --- | --- |
|  | | Wertelabel | N |
| Gruppe | 1 | Junioren Gruppe 1 | 19 |
|  | 2 | Junioren Gruppe 2 | 21 |

| **Deskriptive Statistiken** | | | | |
| --- | --- | --- | --- | --- |
|  | Gruppe | Mittelwert | Std.-Abweichung | N |
| WB_Zeit | Junioren Gruppe 1 | 3,7774 | ,36474 | 19 |
|  | Junioren Gruppe 2 | 4,1751 | ,54191 | 21 |
|  | Gesamt | 3,9862 | ,50245 | 40 |
| NH_Zeit | Junioren Gruppe 1 | 3,8025 | ,54310 | 19 |
|  | Junioren Gruppe 2 | 4,1833 | ,62328 | 21 |
|  | Gesamt | 4,0024 | ,61028 | 40 |
| NHA_Zeit | Junioren Gruppe 1 | 3,7885 | ,45365 | 19 |
|  | Junioren Gruppe 2 | 4,1370 | ,53814 | 21 |
|  | Gesamt | 3,9715 | ,52398 | 40 |
| NB_Zeit | Junioren Gruppe 1 | 3,6476 | ,49945 | 19 |
|  | Junioren Gruppe 2 | 3,9805 | ,55884 | 21 |
|  | Gesamt | 3,8224 | ,55102 | 40 |

| **Mauchly-Test auf Sphärizität^a^** | | | | | | | |
| --- | --- | --- | --- | --- | --- | --- | --- |
| Maß: MEASURE_1 | | | | | | | |
| Innersubjekteffekt | Mauchly-W | Approx. Chi-Quadrat | df | Sig. | Epsilon^b^ | | |
|  |  |  |  |  | Greenhouse-Geisser | Huynh-Feldt | Untergrenze |
| Körpervisualisierung | ,762 | 9,993 | 5 | ,076 | ,867 | ,961 | ,333 |
| Prüft die Nullhypothese, daß sich die Fehlerkovarianz-Matrix der orthonormalisierten transformierten abhängigen Variablen proportional zur Einheitsmatrix verhält. | | | | | | | |
| a. Design: Konstanter Term + Gruppe  Innersubjektdesign: Körpervisualisierung | | | | | | | |
| b. Kann zum Korrigieren der Freiheitsgrade für die gemittelten Signifikanztests verwendet werden. In der Tabelle mit den Tests der Effekte innerhalb der Subjekte werden korrigierte Tests angezeigt. | | | | | | | |

| **Tests der Innersubjekteffekte** | | | | | | | |
| --- | --- | --- | --- | --- | --- | --- | --- |
| Maß: MEASURE_1 | | | | | | | |
| Quelle | | Quadratsumme vom Typ III | df | Mittel der Quadrate | F | Sig. | Partielles Eta-Quadrat |
| Körpervisualisierung | Sphärizität angenommen | ,816 | 3 | ,272 | 4,359 | ,006 | ,103 |
|  | Greenhouse-Geisser | ,816 | 2,601 | ,314 | 4,359 | ,009 | ,103 |
|  | Huynh-Feldt | ,816 | 2,882 | ,283 | 4,359 | ,007 | ,103 |
|  | Untergrenze | ,816 | 1,000 | ,816 | 4,359 | ,044 | ,103 |
| Körpervisualisierung * Gruppe | Sphärizität angenommen | ,026 | 3 | ,009 | ,140 | ,936 | ,004 |
|  | Greenhouse-Geisser | ,026 | 2,601 | ,010 | ,140 | ,915 | ,004 |
|  | Huynh-Feldt | ,026 | 2,882 | ,009 | ,140 | ,931 | ,004 |
|  | Untergrenze | ,026 | 1,000 | ,026 | ,140 | ,710 | ,004 |
| Fehler(Körpervisualisierung) | Sphärizität angenommen | 7,111 | 114 | ,062 |  |  |  |
|  | Greenhouse-Geisser | 7,111 | 98,825 | ,072 |  |  |  |
|  | Huynh-Feldt | 7,111 | 109,522 | ,065 |  |  |  |
|  | Untergrenze | 7,111 | 38,000 | ,187 |  |  |  |

| **Tests der Zwischensubjekteffekte** | | | | | | |
| --- | --- | --- | --- | --- | --- | --- |
| Maß: MEASURE_1 | | | | | | |
| Transformierte Variable: Mittel | | | | | | |
| Quelle | Quadratsumme vom Typ III | df | Mittel der Quadrate | F | Sig. | Partielles Eta-Quadrat |
| Konstanter Term | 2473,144 | 1 | 2473,144 | 2726,614 | ,000 | ,986 |
| Gruppe | 5,315 | 1 | 5,315 | 5,860 | ,020 | ,134 |
| Fehler | 34,467 | 38 | ,907 |  |  |  |

| **Paarweise Vergleiche** | | | | | | |
| --- | --- | --- | --- | --- | --- | --- |
| Maß: MEASURE_1 | | | | | | |
| (I)Körpervisualisierung | (J)Körpervisualisierung | Mittlere Differenz (I-J) | Standard Fehler | Sig.^b^ | 95% Konfidenzintervall für die Differenz^b^ | |
|  |  |  |  |  | Untergrenze | Obergrenze |
| 1 | 2 | -,017 | ,067 | 1,000 | -,204 | ,170 |
|  | 3 | ,013 | ,053 | 1,000 | -,134 | ,161 |
|  | 4 | ,162^*^ | ,057 | ,045 | ,002 | ,322 |
| 2 | 1 | ,017 | ,067 | 1,000 | -,170 | ,204 |
|  | 3 | ,030 | ,058 | 1,000 | -,131 | ,191 |
|  | 4 | ,179^*^ | ,056 | ,017 | ,022 | ,335 |
| 3 | 1 | -,013 | ,053 | 1,000 | -,161 | ,134 |
|  | 2 | -,030 | ,058 | 1,000 | -,191 | ,131 |
|  | 4 | ,149^*^ | ,040 | ,004 | ,037 | ,261 |
| 4 | 1 | -,162^*^ | ,057 | ,045 | -,322 | -,002 |
|  | 2 | -,179^*^ | ,056 | ,017 | -,335 | -,022 |
|  | 3 | -,149^*^ | ,040 | ,004 | -,261 | -,037 |
| Basiert auf den geschätzten Randmitteln | | | | | | |
| *. Die mittlere Differenz ist auf dem ,05-Niveau signifikant. | | | | | | |
| b. Anpassung für Mehrfachvergleiche: Bonferroni. | | | | | | |

| **3. Gruppe * Körpervisualisierung** | | | | | |
| --- | --- | --- | --- | --- | --- |
| Maß: MEASURE_1 | | | | | |
| Gruppe | Körpervisualisierung | Mittelwert | Standard Fehler | 95%-Konfidenzintervall | |
|  |  |  |  | Untergrenze | Obergrenze |
| Junioren Gruppe 1 | 1 | 3,777 | ,107 | 3,561 | 3,994 |
|  | 2 | 3,802 | ,135 | 3,530 | 4,075 |
|  | 3 | 3,789 | ,115 | 3,556 | 4,021 |
|  | 4 | 3,648 | ,122 | 3,401 | 3,894 |
| Junioren Gruppe 2 | 1 | 4,175 | ,102 | 3,969 | 4,381 |
|  | 2 | 4,183 | ,128 | 3,924 | 4,442 |
|  | 3 | 4,137 | ,109 | 3,916 | 4,358 |
|  | 4 | 3,980 | ,116 | 3,746 | 4,215 |


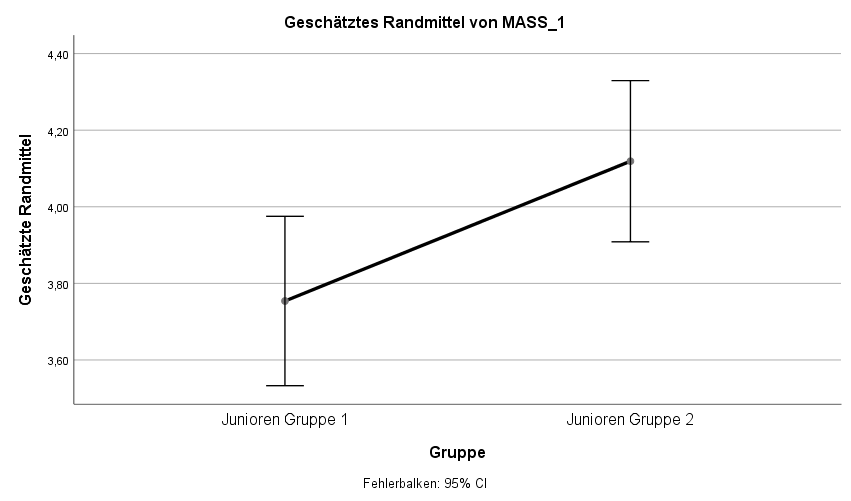

Supplement: S1 Data — (ZIP) [file pone.0263112.s001.zip › Data/Young1vsYoung2/Grasping/GREIFEN ZEIT.docx]
