# Supplementary material for: Influence of body visualization in VR during the execution of motoric tasks in different age groups
Source: PLoS One. 2022 Jan 25;17(1):e0263112. doi: 10.1371/journal.pone.0263112 (PMC8789136; doi:10.1371/journal.pone.0263112)

Throwing Punkte

| **Innersubjektfaktoren** | |
| --- | --- |
| Maß: MEASURE_1 | |
| Körpervisualisierung | Abhängige Variable |
| 1 | WB_P |
| 2 | NHA_P |
| 3 | NB_P |

| **Zwischensubjektfaktoren** | | | |
| --- | --- | --- | --- |
|  | | Wertelabel | N |
| Gruppe | 1 | Junioren Gruppe 1 | 20 |
|  | 2 | Junioren Gruppe 2 | 21 |

| **Deskriptive Statistiken** | | | | |
| --- | --- | --- | --- | --- |
|  | Gruppe | Mittelwert | Std.-Abweichung | N |
| WB_P | Junioren Gruppe 1 | 1,6833 | ,54585 | 20 |
|  | Junioren Gruppe 2 | 1,6986 | ,50447 | 21 |
|  | Gesamt | 1,6911 | ,51849 | 41 |
| NHA_P | Junioren Gruppe 1 | 1,4167 | ,51725 | 20 |
|  | Junioren Gruppe 2 | 1,4763 | ,50086 | 21 |
|  | Gesamt | 1,4472 | ,50341 | 41 |
| NB_P | Junioren Gruppe 1 | 1,3167 | ,58714 | 20 |
|  | Junioren Gruppe 2 | 1,3967 | ,58330 | 21 |
|  | Gesamt | 1,3576 | ,57923 | 41 |

| **Mauchly-Test auf Sphärizität^a^** | | | | | | | |
| --- | --- | --- | --- | --- | --- | --- | --- |
| Maß: MEASURE_1 | | | | | | | |
| Innersubjekteffekt | Mauchly-W | Approx. Chi-Quadrat | df | Sig. | Epsilon^b^ | | |
|  |  |  |  |  | Greenhouse-Geisser | Huynh-Feldt | Untergrenze |
| Körpervisualisierung | ,904 | 3,847 | 2 | ,146 | ,912 | ,979 | ,500 |
| Prüft die Nullhypothese, daß sich die Fehlerkovarianz-Matrix der orthonormalisierten transformierten abhängigen Variablen proportional zur Einheitsmatrix verhält. | | | | | | | |
| a. Design: Konstanter Term + Gruppe  Innersubjektdesign: Körpervisualisierung | | | | | | | |
| b. Kann zum Korrigieren der Freiheitsgrade für die gemittelten Signifikanztests verwendet werden. In der Tabelle mit den Tests der Effekte innerhalb der Subjekte werden korrigierte Tests angezeigt. | | | | | | | |

| **Tests der Innersubjekteffekte** | | | | | | | |
| --- | --- | --- | --- | --- | --- | --- | --- |
| Maß: MEASURE_1 | | | | | | | |
| Quelle | | Quadratsumme vom Typ III | df | Mittel der Quadrate | F | Sig. | Partielles Eta-Quadrat |
| Körpervisualisierung | Sphärizität angenommen | 2,453 | 2 | 1,226 | 7,475 | ,001 | ,161 |
|  | Greenhouse-Geisser | 2,453 | 1,824 | 1,344 | 7,475 | ,002 | ,161 |
|  | Huynh-Feldt | 2,453 | 1,958 | 1,253 | 7,475 | ,001 | ,161 |
|  | Untergrenze | 2,453 | 1,000 | 2,453 | 7,475 | ,009 | ,161 |
| Körpervisualisierung * Gruppe | Sphärizität angenommen | ,022 | 2 | ,011 | ,068 | ,934 | ,002 |
|  | Greenhouse-Geisser | ,022 | 1,824 | ,012 | ,068 | ,920 | ,002 |
|  | Huynh-Feldt | ,022 | 1,958 | ,011 | ,068 | ,931 | ,002 |
|  | Untergrenze | ,022 | 1,000 | ,022 | ,068 | ,795 | ,002 |
| Fehler(Körpervisualisierung) | Sphärizität angenommen | 12,797 | 78 | ,164 |  |  |  |
|  | Greenhouse-Geisser | 12,797 | 71,149 | ,180 |  |  |  |
|  | Huynh-Feldt | 12,797 | 76,371 | ,168 |  |  |  |
|  | Untergrenze | 12,797 | 39,000 | ,328 |  |  |  |

| **Tests der Zwischensubjekteffekte** | | | | | | |
| --- | --- | --- | --- | --- | --- | --- |
| Maß: MEASURE_1 | | | | | | |
| Transformierte Variable: Mittel | | | | | | |
| Quelle | Quadratsumme vom Typ III | df | Mittel der Quadrate | F | Sig. | Partielles Eta-Quadrat |
| Konstanter Term | 275,864 | 1 | 275,864 | 502,533 | ,000 | ,928 |
| Gruppe | ,082 | 1 | ,082 | ,149 | ,701 | ,004 |
| Fehler | 21,409 | 39 | ,549 |  |  |  |

| **Paarweise Vergleiche** | | | | | | |
| --- | --- | --- | --- | --- | --- | --- |
| Maß: MEASURE_1 | | | | | | |
| (I)Körpervisualisierung | (J)Körpervisualisierung | Mittlere Differenz (I-J) | Standard Fehler | Sig.^b^ | 95% Konfidenzintervall für die Differenz^b^ | |
|  |  |  |  |  | Untergrenze | Obergrenze |
| 1 | 2 | ,244^*^ | ,095 | ,042 | ,007 | ,482 |
|  | 3 | ,334^*^ | ,074 | ,000 | ,148 | ,520 |
| 2 | 1 | -,244^*^ | ,095 | ,042 | -,482 | -,007 |
|  | 3 | ,090 | ,097 | 1,000 | -,153 | ,333 |
| 3 | 1 | -,334^*^ | ,074 | ,000 | -,520 | -,148 |
|  | 2 | -,090 | ,097 | 1,000 | -,333 | ,153 |
| Basiert auf den geschätzten Randmitteln | | | | | | |
| *. Die mittlere Differenz ist auf dem ,05-Niveau signifikant. | | | | | | |
| b. Anpassung für Mehrfachvergleiche: Bonferroni. | | | | | | |


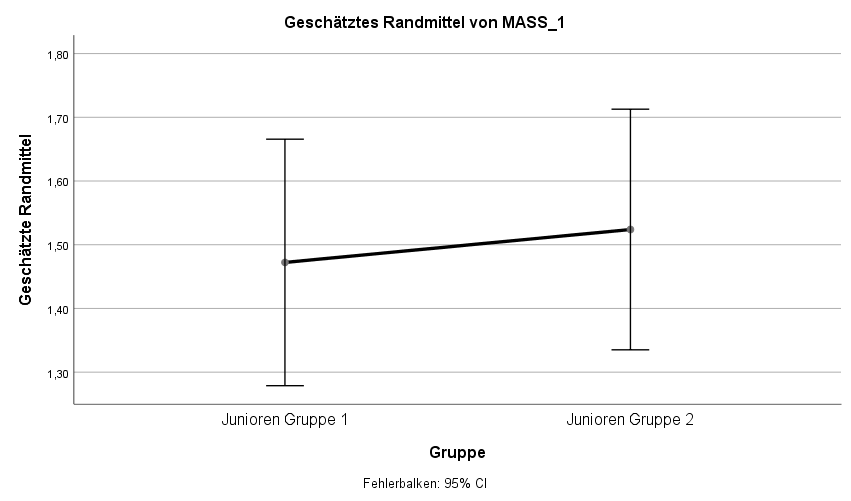

Supplement: S1 Data — (ZIP) [file pone.0263112.s001.zip › Data/Young1vsYoung2/Throwing/Throwing Punkte.docx]
