# Supplementary material for: Influence of body visualization in VR during the execution of motoric tasks in different age groups
Source: PLoS One. 2022 Jan 25;17(1):e0263112. doi: 10.1371/journal.pone.0263112 (PMC8789136; doi:10.1371/journal.pone.0263112)

THROWING SE

| **Innersubjektfaktoren** | |
| --- | --- |
| Maß: MEASURE_1 | |
| Körpervisualisierung | Abhängige Variable |
| 1 | WB_SE |
| 2 | NHA_SE |
| 3 | NB_SE |

| **Zwischensubjektfaktoren** | | | |
| --- | --- | --- | --- |
|  | | Wertelabel | N |
| Gruppe | 1 | Junioren Gruppe 1 | 21 |
|  | 2 | Junioren Gruppe 2 | 20 |

| **Mauchly-Test auf Sphärizität^a^** | | | | | | | |
| --- | --- | --- | --- | --- | --- | --- | --- |
| Maß: MEASURE_1 | | | | | | | |
| Innersubjekteffekt | Mauchly-W | Approx. Chi-Quadrat | df | Sig. | Epsilon^b^ | | |
|  |  |  |  |  | Greenhouse-Geisser | Huynh-Feldt | Untergrenze |
| Körpervisualisierung | ,963 | 1,430 | 2 | ,489 | ,964 | 1,000 | ,500 |
| Prüft die Nullhypothese, daß sich die Fehlerkovarianz-Matrix der orthonormalisierten transformierten abhängigen Variablen proportional zur Einheitsmatrix verhält. | | | | | | | |
| a. Design: Konstanter Term + Gruppe  Innersubjektdesign: Körpervisualisierung | | | | | | | |
| b. Kann zum Korrigieren der Freiheitsgrade für die gemittelten Signifikanztests verwendet werden. In der Tabelle mit den Tests der Effekte innerhalb der Subjekte werden korrigierte Tests angezeigt. | | | | | | | |

| **Tests der Innersubjekteffekte** | | | | | | | |
| --- | --- | --- | --- | --- | --- | --- | --- |
| Maß: MEASURE_1 | | | | | | | |
| Quelle | | Quadratsumme vom Typ III | df | Mittel der Quadrate | F | Sig. | Partielles Eta-Quadrat |
| Körpervisualisierung | Sphärizität angenommen | 7,993 | 2 | 3,996 | 17,470 | ,000 | ,309 |
|  | Greenhouse-Geisser | 7,993 | 1,929 | 4,144 | 17,470 | ,000 | ,309 |
|  | Huynh-Feldt | 7,993 | 2,000 | 3,996 | 17,470 | ,000 | ,309 |
|  | Untergrenze | 7,993 | 1,000 | 7,993 | 17,470 | ,000 | ,309 |
| Körpervisualisierung * Gruppe | Sphärizität angenommen | ,004 | 2 | ,002 | ,008 | ,992 | ,000 |
|  | Greenhouse-Geisser | ,004 | 1,929 | ,002 | ,008 | ,991 | ,000 |
|  | Huynh-Feldt | ,004 | 2,000 | ,002 | ,008 | ,992 | ,000 |
|  | Untergrenze | ,004 | 1,000 | ,004 | ,008 | ,930 | ,000 |
| Fehler(Körpervisualisierung) | Sphärizität angenommen | 17,843 | 78 | ,229 |  |  |  |
|  | Greenhouse-Geisser | 17,843 | 75,221 | ,237 |  |  |  |
|  | Huynh-Feldt | 17,843 | 78,000 | ,229 |  |  |  |
|  | Untergrenze | 17,843 | 39,000 | ,458 |  |  |  |

| **Tests der Zwischensubjekteffekte** | | | | | | |
| --- | --- | --- | --- | --- | --- | --- |
| Maß: MEASURE_1 | | | | | | |
| Transformierte Variable: Mittel | | | | | | |
| Quelle | Quadratsumme vom Typ III | df | Mittel der Quadrate | F | Sig. | Partielles Eta-Quadrat |
| Konstanter Term | 1339,897 | 1 | 1339,897 | 180,105 | ,000 | ,822 |
| Gruppe | ,125 | 1 | ,125 | ,017 | ,898 | ,000 |
| Fehler | 290,142 | 39 | 7,440 |  |  |  |

| **Paarweise Vergleiche** | | | | | | |
| --- | --- | --- | --- | --- | --- | --- |
| Maß: MEASURE_1 | | | | | | |
| (I)Körpervisualisierung | (J)Körpervisualisierung | Mittlere Differenz (I-J) | Standard Fehler | Sig.^b^ | 95% Konfidenzintervall für die Differenz^b^ | |
|  |  |  |  |  | Untergrenze | Obergrenze |
| 1 | 2 | -,577^*^ | ,107 | ,000 | -,846 | -,308 |
|  | 3 | -,496^*^ | ,113 | ,000 | -,779 | -,213 |
| 2 | 1 | ,577^*^ | ,107 | ,000 | ,308 | ,846 |
|  | 3 | ,081 | ,096 | 1,000 | -,158 | ,321 |
| 3 | 1 | ,496^*^ | ,113 | ,000 | ,213 | ,779 |
|  | 2 | -,081 | ,096 | 1,000 | -,321 | ,158 |
| Basiert auf den geschätzten Randmitteln | | | | | | |
| *. Die mittlere Differenz ist auf dem ,05-Niveau signifikant. | | | | | | |
| b. Anpassung für Mehrfachvergleiche: Bonferroni. | | | | | | |


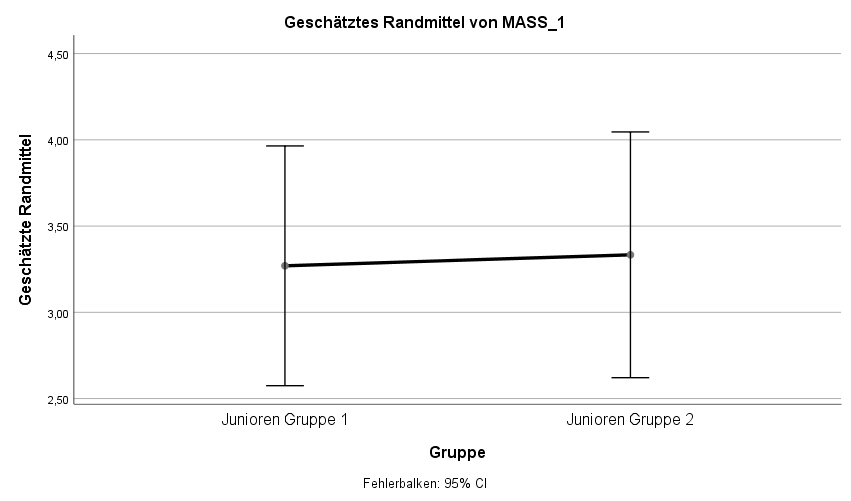

Supplement: S1 Data — (ZIP) [file pone.0263112.s001.zip › Data/Young1vsYoung2/Throwing/THROWING SE.docx]
