# Supplementary figures and images for: Influence of body visualization in VR during the execution of motoric tasks in different age groups
Source: PLoS One. 2022 Jan 25;17(1):e0263112. doi: 10.1371/journal.pone.0263112 (PMC8789136; doi:10.1371/journal.pone.0263112)

## Slide 1
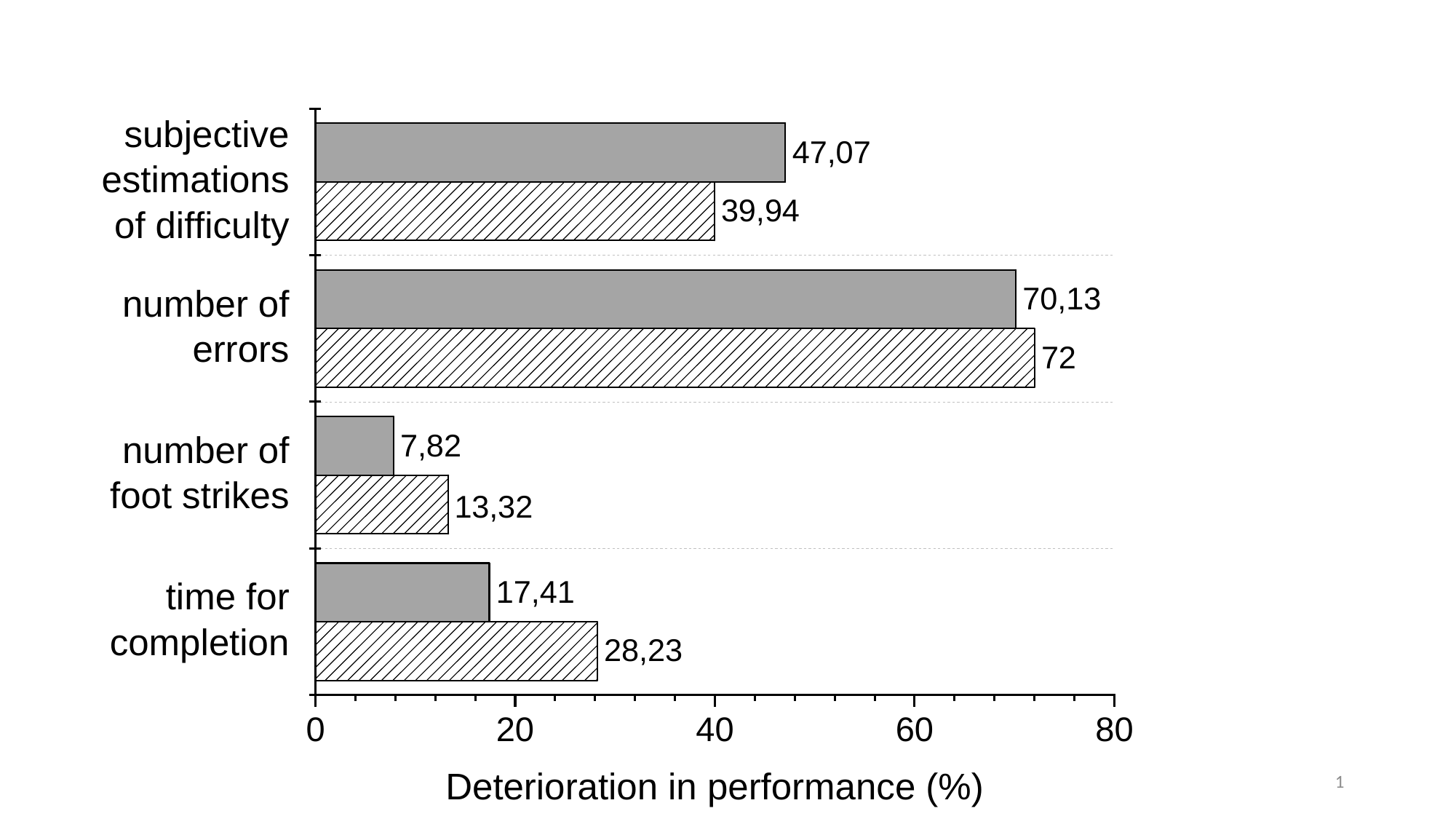

1

Supplement: S1 Data — (ZIP) [file pone.0263112.s001.zip › Data/Young2vsOld/Balance task.pptx]

## Slide 1
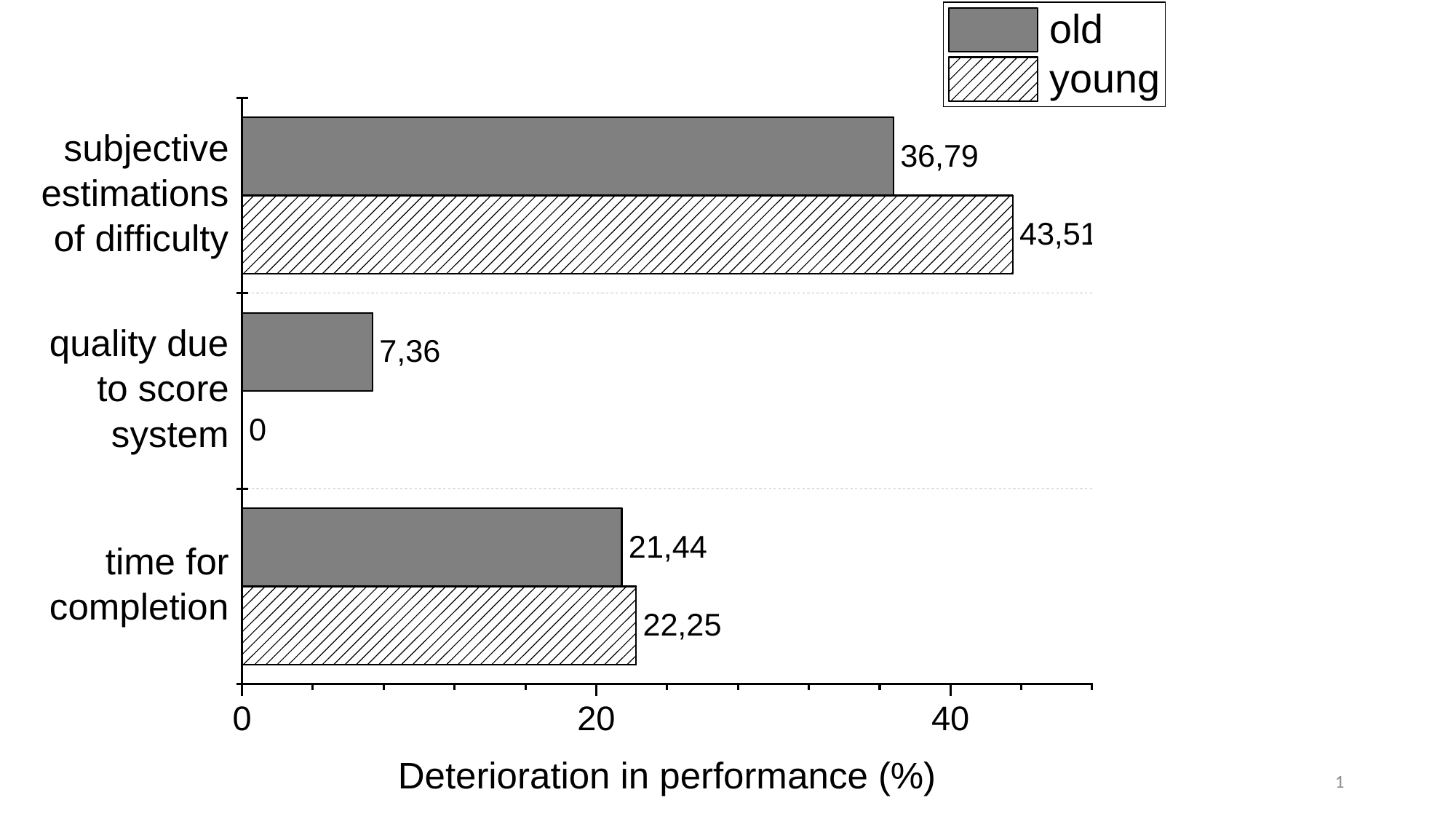

1

Supplement: S1 Data — (ZIP) [file pone.0263112.s001.zip › Data/Young2vsOld/Grasping.pptx]

## Slide 1
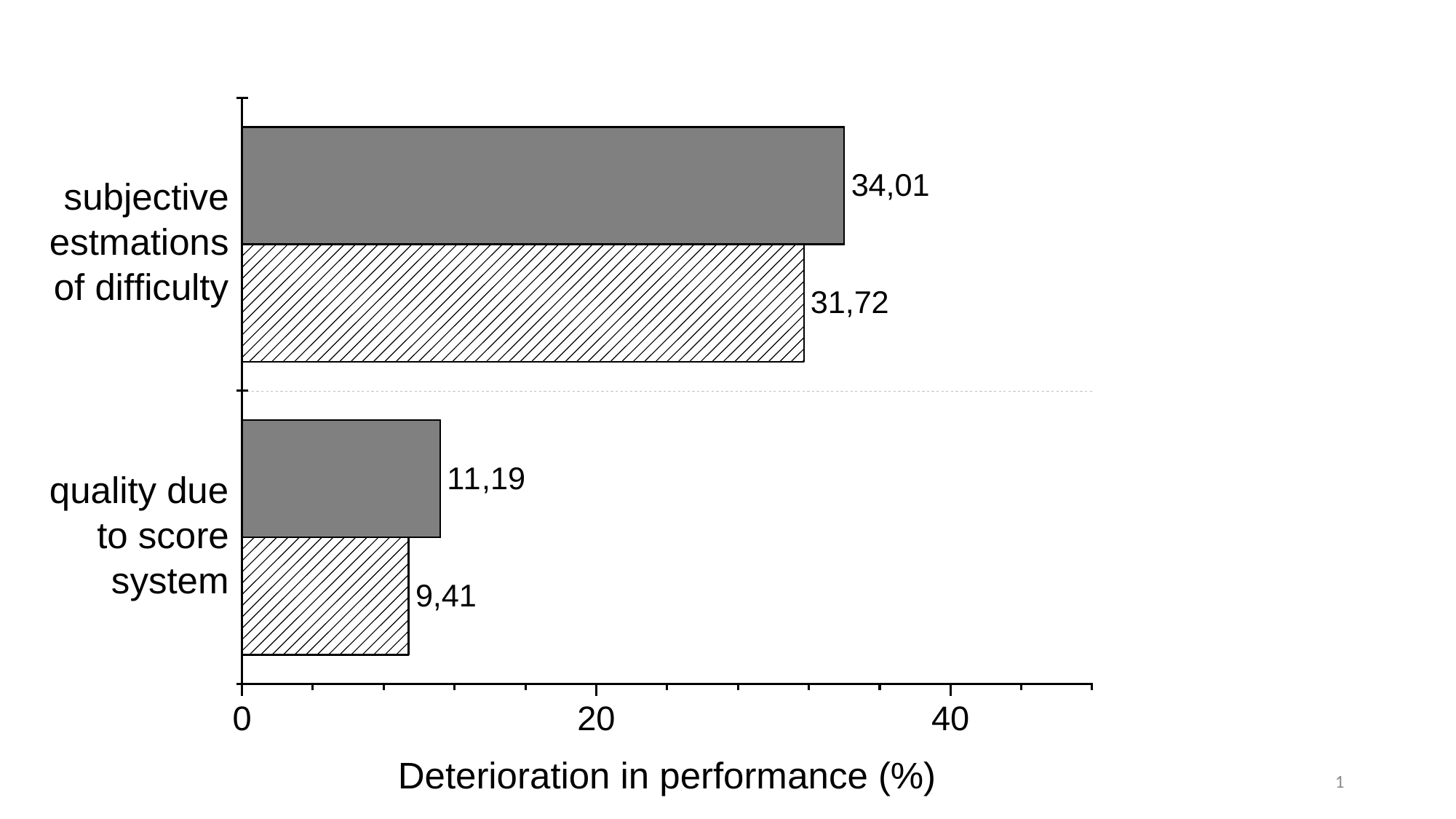

1

Supplement: S1 Data — (ZIP) [file pone.0263112.s001.zip › Data/Young2vsOld/Throwing.pptx]
